# Uncovering the trimethylamine-producing bacteria of the human gut microbiota

**DOI:** 10.1186/s40168-017-0271-9

**Published:** 2017-05-15

**Authors:** Silke Rath, Benjamin Heidrich, Dietmar H. Pieper, Marius Vital

**Affiliations:** 1 Microbial Interactions and Processes Research Group, Helmholtz Centre for Infection Research, Inhoffenstraße 7, Braunschweig, 38124 Germany; 2 Department of Gastroenterology, Hepatology and Endocrinology, Hannover Medical School, Hannover, Germany

**Keywords:** Trimethylamine, Gut microbiota, Microbiome, Atherosclerosis, Cardiovascular disease, Functional diagnostics

## Abstract

**Background:**

Trimethylamine (TMA), produced by the gut microbiota from dietary quaternary amines (mainly choline and carnitine), is associated with atherosclerosis and severe cardiovascular disease. Currently, little information on the composition of TMA producers in the gut is available due to their low abundance and the requirement of specific functional-based detection methods as many taxa show disparate abilities to produce that compound.

**Results:**

In order to examine the TMA-forming potential of microbial communities, we established databases for the key genes of the main TMA-synthesis pathways, encoding choline TMA-lyase (*cutC*) and carnitine oxygenase (*cntA*), using a multi-level screening approach on 67,134 genomes revealing 1107 and 6738 candidates to exhibit *cutC* and *cntA*, respectively. Gene-targeted assays enumerating the TMA-producing community by quantitative PCR and characterizing its composition via Illumina sequencing were developed and applied on human fecal samples (*n* = 50) where all samples contained potential TMA producers (*cutC* was detected in all individuals, whereas only 26% harbored *cntA*) constituting, however, only a minor part of the total community (below 1% in most samples). Obtained c*utC* amplicons were associated with various taxa, in particular with *Clostridium* XIVa strains and *Eubacterium* sp. strain AB3007, though a bulk of sequences displayed low nucleotide identities to references (average 86% ± 7%) indicating that key human TMA producers are yet to be isolated. Co-occurrence analysis revealed specific groups governing the community structure of *cutC*-exhibiting taxa across samples. *CntA* amplicons displayed high identities (~99%) to *Gammaproteobacteria*-derived references, primarily from *Escherichia coli*. Metagenomic analysis of samples provided by the Human Microbiome Project (*n* = 154) confirmed the abundance patterns as well as overall taxonomic compositions obtained with our assays, though at much lower resolution, whereas 16S ribosomal RNA gene sequence analysis could not adequately uncover the TMA-producing potential.

**Conclusions:**

In this study, we developed a diagnostic framework that enabled the quantification and comprehensive characterization of the TMA-producing potential in human fecal samples. The key players were identified, and together with predictions on their environmental niches using functional genomics on most closely related reference strains, we provide crucial information for the development of specific treatment strategies to restrain TMA producers and limit their proliferation.

**Electronic supplementary material:**

The online version of this article (doi:10.1186/s40168-017-0271-9) contains supplementary material, which is available to authorized users.

## Background

The human body hosts a myriad of bacteria that play important roles in host health and disease. It is becoming evident that besides particular pathogens, whole microbial consortia are involved in the development of certain diseases. This is exemplified by trimethylamine (TMA), a microbial metabolite that is produced by various taxa of the gut microbiota from dietary quaternary amines, mainly choline and carnitine. TMA is absorbed via the intestinal epithelium and further oxidized to trimethylamine *N*-oxide (TMAO), which was associated with atherosclerosis and severe cardiovascular disease in several independent studies [1–4]. A mechanistic model has been formulated proposing that TMAO promotes atherogenesis through the formation of foam cells (lipid-laden macrophages) and a reduction of the reverse cholesterol transport from the atherosclerotic plaque [5].

Two major TMA-synthesis pathways have been described with a specific glycyl radical enzyme, the choline TMA-lyase (CutC) and its activator CutD [6], that takes choline as a substrate and a two-component Rieske-type oxygenase/reductase (CntA/B) [7], which acts on carnitine and its derivative gamma-butyrobetaine, as the key enzymes. An additional enzyme complex termed YeaW/X, which shows close sequence similarity to CntA/B, was recently proposed as the key component of a third major pathway [8]. Campo and colleagues have shown that the so-called choline utilization cluster, which includes *cutC/D* among other genes involved in anaerobic choline metabolism, is widely, but discontinuously, distributed across various taxa belonging to *Firmicutes*, *Actinobacteria*, and *Proteobacteria* [9]. The authors biochemically verified TMA synthesis from choline for a multitude of the revealed taxa and demonstrated that both *cutC* and *cutD* are required for the TMA-generating cleavage reaction. Another recent study [10] demonstrated that colonization of the mouse gut with a specific consortium of CutC-encoding human isolates results in TMA synthesis and subsequent TMAO accumulation in the serum of animals where even minute concentrations of TMA producers (0.15% of the total community) were sufficient for substantial TMA production from choline. Genes encoding CntA/B and YeaX/Y, respectively, have been described in several taxa from the *Gamma*- and *Betaproteobacteria* as well as from a few *Firmicutes* [7]. Functionality has been shown for *Acinetobacter baumannii* and *Escherichia coli* where both genes *cntA/yeaX* and *cntB/yeaW* are required for the formation of TMA.

Despite this multitude of knowledge on TMA-producing bacteria and their importance for human health, little information on their abundance and taxonomic composition in vivo is available. So far, only one study estimates the overall potential of intestinal communities to produce TMA [11], and specific structural insights into this functional community are largely lacking. This can partly be explained by the low abundance of TMA producers and the requirement of specific methods for their quantification, as phylogenetic markers are expected to be poor predictors for that function, at least for the broadly polyphyletic bacteria exhibiting *cutC/D* [9]. Thus, the aim of this study was to build a comprehensive framework in order to quantify the TMA-production potential of intestinal communities and to gain detailed compositional insights into this important functional group. To this end, comprehensive databases for the key genes of all major pathways were constructed and gene-targeted assays were designed for quantitative PCR (qPCR) coupled to sequencing of PCR products on the Illumina MiSeq platform. The developed assays were used to characterize the TMA-producing communities in the fecal samples of 50 individuals.

## Results

### Establishing databases for *cutC* and *cntA*

Since any gene-based investigations depend on reference sequences, the entire diversity spectrum of the key genes from major pathways involved in the formation of TMA was identified. Comprehensive databases for *cutC* and *cntA* were established applying a multi-level screening approach on 67,134 genomes provided by the Pathosystems Resource Integration Center (PATRIC). *CntA* and *yeaW* show high similarity, and sequences from both genes were included in our reference set used to construct a single hidden Markov model (HMM) for this gene group. All genes comprised in this database will be referred to as *cntA* throughout the study (see also the “Discussion” section).

**Fig. 1:**
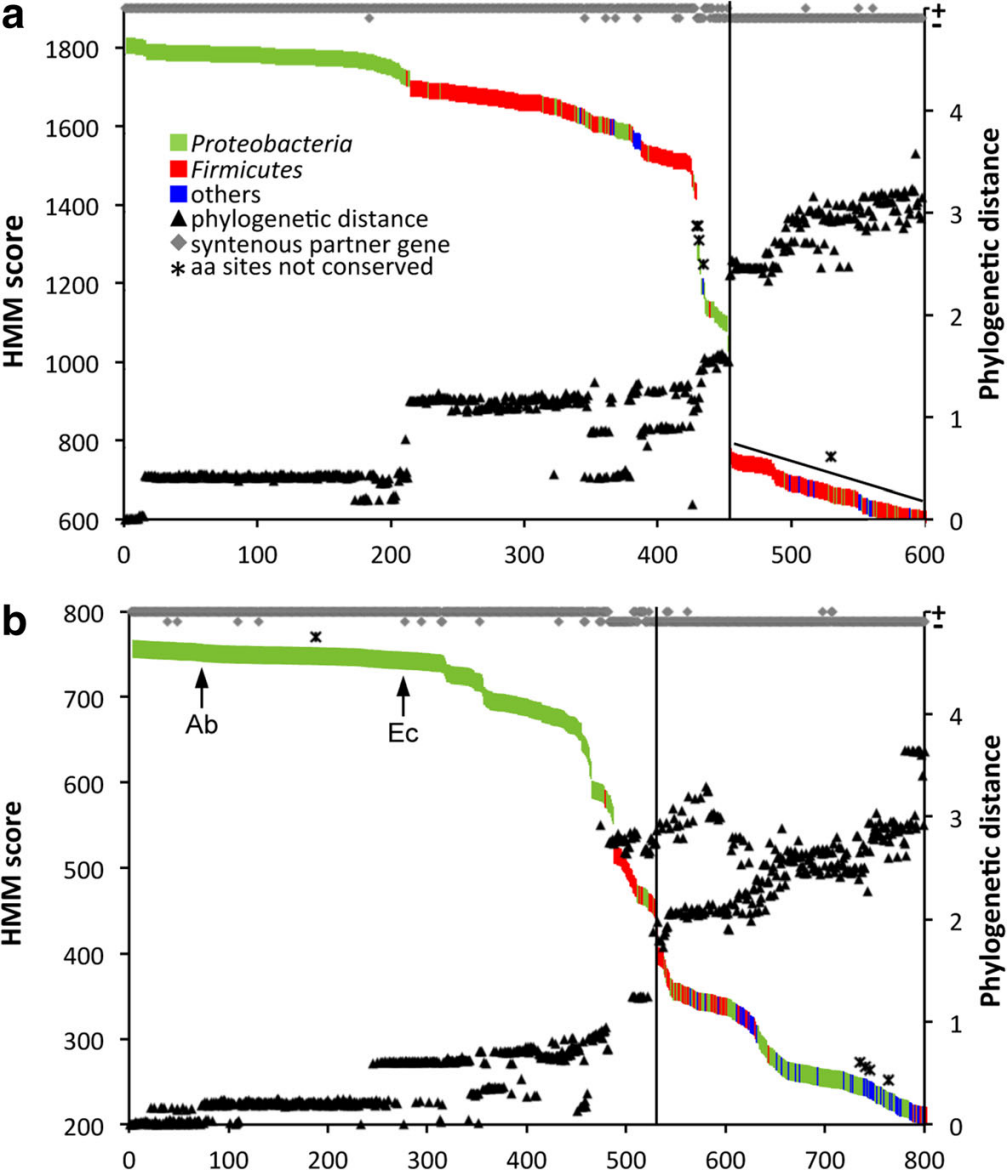
Results of the multiparametric genome-screening approach for the genes *cutC* (**a**) and *cntA* (**b**). Obtained unique proteins are depicted along the *horizontal axis* where they are sorted according to their similarity to the constructed hidden Markov chain models (HMM) represented by the *primary vertical axis*. The *secondary vertical axis* depicts both the phylogenetic distance to the top-scoring sequences (*triangles*) and synteny (*diamonds*) to the corresponding partner genes *cutD* and *cntB*. Taxonomic affiliations of sequences are indicated by the *color code. Asterisks* indicate sequences lacking conserved amino acid residues. Cutoffs set to discriminate true *cutC* (HMM score of 906.4) and *cntA* (HMM score of 440.6) genes are illustrated in the respective plots. For *cntA*, phylogeny was applied as an additional filtering step (see text). Final databases for *cutC* and *cntA* contained 1114 (454 unique proteins) and 6772 (491 unique proteins) candidate genes, respectively. *CntA* reference sequences derived from *Acinetobacter baumannii* (*Ab*) and *Escherichia coli* (*Ec*; previously termed *yeaW*) are highlighted by *arrows*

Figure 1 illustrates the result of the multi-level genome-screening approach considering the following three criteria: (i) similarity to developed HMMs, (ii) conservation of specific amino acid sites previously suggested as signatures for respective encoding genes [6, 7], and (iii) phylogenetic distance to top-scoring sequences. Additionally, synteny with the associated activator gene (*cutD*) as well as the reductase (*cntB*) that were both shown to be essential for TMA production in the corresponding pathways was investigated. Putative *cutC* genes were detected in a total of 1107 genomes (454 dereplicated protein sequences) that belong to *Proteobacteria* (in particular *Gamma*- and *Deltaproteobacteria*) and *Firmicutes* (mainly *Clostridia* and some *Bacilli*), as well as a few *Actinobacteria* (Fig. 1a, Additional file 1A, Additional file 2A). A clear drop in the HMM similarity score was detected after sequence 453 that co-occurred with abrupt changes in other parameters investigated including phylogenetic distance to the top-scoring sequence depicting a profound change in gene sequences below that drop. Hence, a cutoff of 906.4, representing the median HMM score of the two sequences that frame that drop, was set, and all entries above that cutoff were considered as true *cutC* sequences. With a few exceptions, they all show synteny with *cutD* and display conserved amino acid residues previously suggested to be characteristic for the respective gene product [6]. Our results are in line with phylogeny where *cutC* sequences form a cluster apart from sequences below our set HMM cutoff (Additional file 1C). All revealed candidates contain a single copy of *cutC*, except for seven genomes that display double entries.

Putative *cntA* genes were identified in 6738 genomes (491 dereplicated protein sequences) where the majority was observed in *Proteobacteria*, especially *Gammaproteobacteria* (mostly derived from *Escherichia* and *Acinetobacter*), and some entries belonging to *Betaproteobacteria* and *Epsilonproteobacteria* (Fig. 1b, Additional file 1B, Additional file 2B). Two sequences were obtained from *Enterococcus* sp. Similar to *cutC*, the chosen HMM similarity cutoff was set at an obvious HMM score drop that, however, correlated less strongly with other parameters investigated in comparison with the *cutC* analysis shown above. Specifically, 34 lower-scoring sequences from *Bacilli* are neither in close phylogenetic relation to the top-scoring sequences nor do they exhibit syntenic *cntB* sequences and were, hence, not considered as true *cntA* (see also Additional file 1D)*.* All sequences (with one exception) display a conserved “bridging” glutamate instead of aspartate (E205D), which was previously suggested as a signature for that enzyme [7]. Revealed candidates exhibit *cntA* as a single copy gene, except for 31 genomes, mainly *A. baumannii* and *E. coli* strains, where more than one target was detected. However, the majority of those sequences were identical to other *cntA* genes in the respective genomes, and only two candidates (*Klebsiella pneumoniae* BIDMC 18C and *Cupriavidus taiwanensis* STM 6070) exhibited two non-identical *cntA* sequences.

### Gene-targeted assays to investigate the TMA-producing community

Gene-targeted assays for *cutC* and *cntA* were developed in order to determine the potential of the gut microbiota to produce TMA and to characterize the composition of this functional community in detail. In silico analysis on coverage of developed primers suggested that a broad range of *cutC* and *cntA* gene sequences are targeted, namely 93 and 96% of all unique *cutC* and *cntA* genes, respectively (allowing for one mismatch in each primer). To test actual primer performance on various targets in vitro, short synthetic sequences that contained the target sequences from distinct taxa covering a broad thermodynamic range were used as a template in qPCR reactions. For both genes, all tested synthetic sequences were amplified and, with a few exceptions, quantified at similar abundance levels (Additional file 3C, D ). Based on genomic sequences that were used as standards in the developed assays (see the “Methods” section), a quantification limit of 5 × 10^3^ genome copies per reaction for both genes was determined. Below those concentrations, the formation of primer dimers did not allow for accurate target enumeration (Additional file 3A, B).

**Fig. 2:**
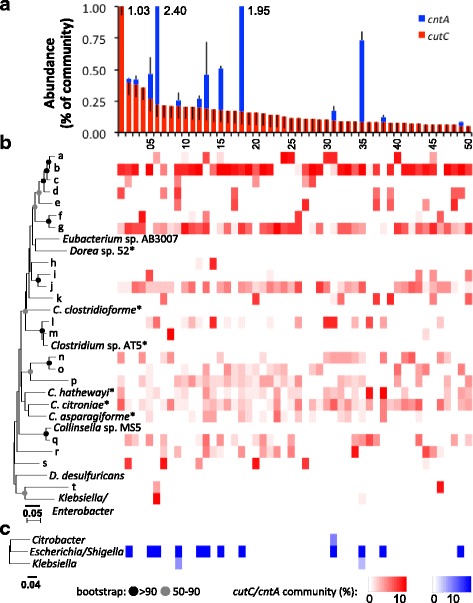
TMA-producing communities of fecal samples revealed by gene-targeting assays. Panel **a** depicts gene abundances of *cutC* (*red*) and *cntA* (*blue*) relative to the total amount of 16S rRNA gene copies of a sample. Volunteers are sorted by descending quantity of *cutC*, and *error bars* represent standard deviation on triplicate measurements. The composition of *cutC* (**b**) and *cntA* (**c**) genes is presented as neighbor-joining trees comprising representative sequences derived from complete-linkage clustering on the nucleotide level using a 90% identity cutoff. Heatmaps are aligned with bars from panel **a** to illustrate the composition of each gene for all samples (relative abundances of clusters are shown). Clusters that contained reference sequences are indicated by name (*Clostridium* sp. AT5 (1720194.3.peg.1841); *C. hathewayi* (566550.8.peg.2440); *Clostridium citroniae* WAL19142 (742734.4.peg.3562)/*Clostridium* sp. FS41; *Clostridium asparagiforme* DSM 15981 (518636.5.peg.3238)/*Clostridiales bacterium* VE202-15 for *cutC* and *Escherichia/Shigella* (1169329.3.peg.2590); *Citrobacter* (742730.3.peg.4068); *Klebsiella* (1308539.3.peg.2257) for *cntA*; specific sequence IDs that were used to build the trees are given in *brackets*), whereas *letters* represent clusters that did not include any reference. C*utC* reference sequences that are closely related to representative sequences of clusters are displayed in the tree as well (*Eubacterium* sp. AB3007 (1392487.3.peg.725); *Dorea* sp. 52 (1235798.3.peg.5630); *Clostridium clostridioforme* AGR2157 (1280695.3.peg.868); *Collinsella* sp. MS5 (1499681.3.peg.1502); *Desulfovibrio desulfuricans* subsp. *desulfuricans* DSM 642 (1121445.4.peg.1354)). Bootstrap values ≥50 and ≥90% are indicated by *circles*. Only major clusters of highest abundances are shown representing 80 and 99% of all *cutC* and *cntA* sequences, respectively. *Asterisks* highlight the reference sequences belonging to *Clostridium* XIVa strains

The developed assays were subsequently applied on 50 human fecal samples (Fig. 2a). *CutC* was enumerated in all samples, and an average abundance of 0.16% ± 0.15% bacteria of the total community was calculated to contain the gene. *CntA* was quantified in 13 samples displaying large abundance differences ranging from 0.03 to 2.17% of bacteria possessing *cntA*, with only three samples displaying an abundance ≥0.5%. To quality control the qPCR results, we performed melting curve analysis for all reactions and separated amplified products on an agarose gel for visual inspections (Additional file 4). For *cutC*, one major band with the expected size was detected in all samples; for a few samples, some very faint unspecific bands were additionally visible. In the *cntA* qPCR, two samples (10 and 19) showed a single unspecific band (sequencing confirmed them as non-target (data not shown)) and samples were, hence, not considered as positive for the gene; one sample displayed two bands. The qPCR on one third diluted samples indicated quantities of 40.6% ± 12.2% and 39.9% ± 22.2% of undiluted samples for *cutC* and *cntA* (five samples were on the borderline of detection), respectively, evidencing that the PCR mixtures were devoid of any interfering ingredients. Gene-abundance results did not significantly correlate with any subject parameters presented in Additional file 5.

In order to characterize the TMA-producing community in detail, amplified products were sequenced on the Illumina MiSeq platform. *CutC* genes similar to those previously observed in all three phyla presented in the database, namely *Actinobacteria*, *Firmicutes*, and *Proteobacteria*, were detected. On average, 76 ± 18 clusters per sample (90% nucleotide identity) were observed, where the majority was similar to genes previously observed in *Firmicutes*, in particular to those of *Clostridium* XIVa strains and *Eubacterium* sp. strain AB3007 (Fig. 2b). However, a major part of sequences, especially those of clusters a–k (Fig. 2b), were only distantly related to reference sequences and formed separate clades on the tree. Consequently, overall nucleotide similarity of *cutC* amplicons to reference sequences was low (86% ± 7%). Detailed analysis applying high clustering identity cutoffs (99 and 98% nucleotide similarity) revealed high diversities of the *cutC* gene with several thousand distinct sequence types that are shared between a few samples (<10%; Additional file 6A). At a cutoff level of 95% 1310 distinct sequence types could be differentiated. Only a few of them (41) were detected in the majority of samples; however, they comprised the bulk of obtained sequences (≥67%). Even at the low identity cutoffs of 90%, the vast amount of clusters was only detected in a minority of samples with many being sample specific.

In contrast, all *cntA* sequences obtained were similar to those previously observed in *Gammaproteobacteria*, in particular from *Escherichia/Shigella* (Fig. 2c); closest match analysis revealed 98.1% being closely related to sequences from *E. coli* (data not shown). A few communities additionally contained *cntA* amplicons similar to those previously detected in *Citrobacter* and *Klebsiella*. Overall, the identity of amplicons to reference sequences was high (99.5% ± 0.5%), and with 3 ± 2 clusters (90% nucleotide identity) detected per sample, the gene diversity was much lower compared to that observed for *cutC*. At higher cutoffs (99 and 98% identity), several thousand clusters were detected and richness drastically declined with decreasing identity cutoffs. For example, only 55 and 14 total clusters were detected at 95 and 90% cutoff values, respectively. Of those, a few clusters (nine and one, respectively) were observed in >90% of all samples comprising the majority of obtained sequences (Additional file 6B).

**Fig. 3:**
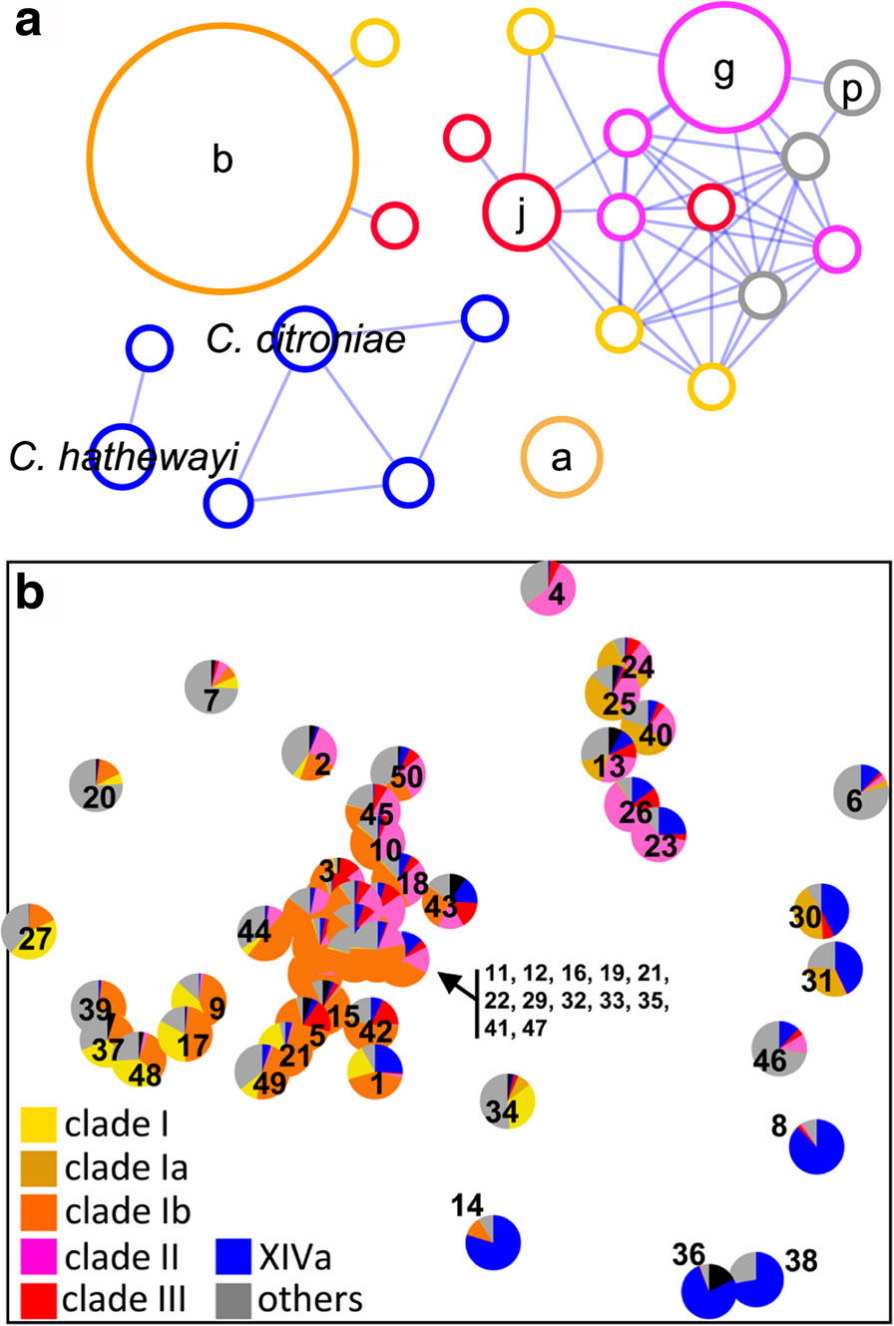
Co-occurrence network and ordination analysis of the *cutC* gene community. Panel **a** displays the co-occurrence network based on the clustering result presented in Fig. 2b. Connected nodes (clusters) are co-occurring (*p* < 0.01, Spearman’s *ρ* ≥ 0.5, false-discovery correction *q*-value <0.01) where only clusters that are detected in at least 50% of samples were considered for analysis. Nodes lacking a *letter* fulfill criteria for co-occurrence analysis, but are not depicted in Fig. 2 due to their low abundance. Node size illustrates relative abundance of the respective cluster. Color codes represent specific clades (clusters a and b from clade I are illustrated as *clade Ia* and *clade Ib*, respectively; *clade II*: cluster g; *clade III*: cluster j; *XIVa*: clusters closely related to *Clostridium* XIVa strains; *others*: clusters related to other taxa) presented in Fig. 2b. Additional clusters that are not shown in Fig. 2 are color-coded according to their respective clades. Panel **b** illustrates non-metric multidimensional scaling analysis (Bray-Curtis dissimilarity) of entire *cutC* gene communities with abundances of clusters depicted in panel **a** highlighted for each sample. *Numbers* refer to corresponding samples shown in Fig. 2

Co-occurrence analysis of *cutC* amplicons based on the clustering results presented in Fig. 2b revealed several distinct network groups that were largely directed by the phylogenetic relatedness of clusters (Fig. 3a). Members of clade I (defined by clusters a–d from Fig. 2b) are separated where the main clusters a and b are not connected to each other nor to other phylogenetically related clusters, whereas members from clades II (g) and III (j) are located in the same module linked to other clusters from those clades. Clusters closely associated with *Clostridium* XIVa strains form separate groups. The observed network patterns were largely reflected in ordination analysis where samples that grouped together in the ordination plot were enriched in specific sequence clusters as expected from co-occurrence analysis (Fig. 3b). In particular, a separation between samples containing either cluster a or b was detected. Samples enriched in other clusters linked to clade I form another group on the plot, whereas a few distant samples displayed high abundances of clusters tightly related with *Clostridium* XIVa strains.

To assess whether taxonomic markers can predict the TMA-production potential of intestinal communities, results from the functional assays were compared to data derived from 16S ribosomal RNA (rRNA) gene sequence analysis. No significant correlations between gene abundances of *cutC* with relative abundances of respective taxa previously described to contain those genes were observed (Additional file 7). Specifically, *Clostridium* XIVa and *Desulfovibrio* relative abundances based on 16S rRNA gene sequence analysis were dissimilar to *cutC* gene abundances obtained with our assay. *Eubacterium* was only detected at very low relative abundances in a few samples. Conversely, in most *cntA*-positive samples (based on qPCR), *Escherichia/Shigella* and *Enterobacteriaceae* were detected by 16S rRNA gene sequence analysis.

### Screening of metagenomes for TMA-producing catabolic genes

**Fig. 4:**
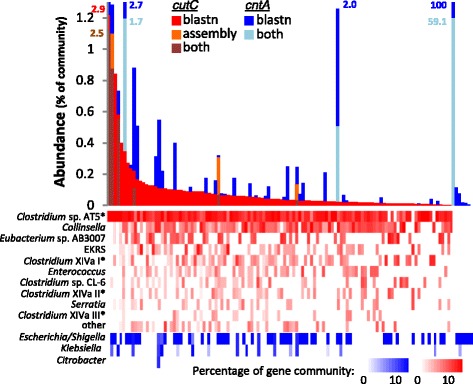
Screening for TMA-producing genes in metagenomes of fecal samples (*n* = 154) from the Human Microbiome Project. On top the relative abundance of bacteria containing *cutC/D* (*red*) and *cntA/B* (*blue*) genes calculated as percentage of the total community indicated by the amount of reads matching the housekeeping gene *rplB* are shown. Samples are sorted along the *horizontal axis* according to their *cutC/D* abundances. Results of the gene-targeted metagenomic assembly method as a fraction of the BLAST (blastn) result are displayed as *dark red* (*n* = 6) and *light blue* (*n* = 3) for *cutC/D* and *cntA/B*, respectively (results for *cutC/D* exceeding those of blastn is shown in *orange*). Samples are sorted by descending quantity of *cutC/D*. Specific numeric labels give values exceeding the maxima of axis. Below associated taxonomic affiliations of respective gene communities are displayed. For *CutC*, reference sequences were subjected to complete-linkage clustering on the nucleotide level based on 90% identity, whereas *cntA* reference sequences were binned on the genus level. The EKRS cluster involves sequences from *Enterobacter*, *Klebsiella*, *Raoultella*, and *Staphylococcus. Clostridium* XIVa I, II, and III refer to distinct clusters composed of *Clostridium* XIVa strains. *Asterisks* highlight sequences derived from *Clostridium* XIVa strains. Samples where no genes encoding TMA production were detected (*n* = 38) are not shown

Metagenomes from 154 samples provided by the Human Microbiome Project were screened for TMA-producing communities. A similar abundance pattern as obtained with the gene-targeted approach was observed with the majority of samples (71%) harboring the key genes of the choline utilization cluster *cutC/D*, though at low concentrations (on average, 0.11% of bacteria were calculated to exhibit the genes), whereas only a minority of samples (27%) were positive for *cntA/B* with a few exhibiting high amounts of those genes (Fig. 4). As shown by amplicon sequencing, the majority of reads indicating the presence of *cutC/D* were similar to genes of *Clostridium* XIVa or *Eubacterium* sp. strain AB3007. However, most sequence reads displayed an identity of only 92.3% ± 7.1% and 96% ± 6.4% for *cutC* and *cutD*, respectively. In contrast, reads indicating the presence of *cntA/B* were nearly identical to references (99.6% ± 1.1% and 99.8% ± 0.8% for *cntA* and *cntB*, respectively) and predominantly related to *Gammaproteobacteria*, in particular to *Escherichia/Shigella* and *Klebsiella*. Application of a gene-targeted assembly method yielded sequences for *cutC/D* and *cntA/B* in only six and three samples, respectively, though at similar abundances as through our BLAST search (Fig. 4). For *cutC/D*, the majority of contigs from those samples were gene fragments and only four assembled sequences (two for each gene) with a length >50% of median reference gene lengths were obtained (from two distinct samples). These sequences were nearly identical (>99%) to those previously observed in *Clostridium* sp. AT5, a *Clostridium* XIVa strain. Screening of contigs provided by the Human Microbiome Project (HMP) for *cutC/D* sequences yielded similar results. Only two samples contained contigs with decent sequence stretches indicating the presence of *cutC/D*, and those were nearly identical (>99% identity) to *Clostridium* sp. AT5 sequences (data not shown). Thus, due to the low abundance of bacteria encoding a TMA-producing potential, sequencing depth was not enough to ensure adequate coverage for assembly (the BLAST searches yielded on average only 56 and 13 reads for *cutC* and *cutD*, respectively), even for HMP datasets where an ample amount of reads (on average >10^8^) is provided.

In order to gain insights into TMA-producing communities of atherosclerotic individuals, we reanalyzed metagenomes provided by Karlsson and colleagues [12], who specifically investigated the microbial composition of symptomatic atherosclerosis patients (*n* = 12) and compared them to healthy control samples (*n* = 13). Our results indicated increased abundances of bacteria exhibiting the potential to produce TMA (by about a factor of 2) in the patient group where the abundances of key genes of both pathways were elevated (median values of 0.39 and 0.11% (patient group) versus 0.22 and 0% (controls) for *cutC* and *cntA*, respectively, as a percentage of bacteria calculated to exhibit the genes) (Additional file 8). However, these observed differences were statistically not significant. Sequencing depth was roughly 1 order of magnitude lower compared to HMP datasets hampering accurate analyses.

### Genomic analyses of *Clostridium* XIVa strains

Ecophysiological insights on key TMA producers and specification of their niches in vivo assist to understand factors that control their abundance and might, eventually, help to design specific treatment strategies for containment of those bacteria. Whereas a wealth of information is available for the main *cntA/B-*exhibiting taxon, *E. coli*, much less is known for members of the diverse genus *Clostridium* XIVa whose *cutC* sequences represent, together with that of *Eubacterium* sp. strain AB3007, the closest known relatives of the majority of obtained amplicons as well as metagenomic reads associated with the gene. Phylogenetic analyses based on 16S rRNA gene sequence similarities demonstrated CutC-encoding *Clostridium* XIVa strains as a polyphyletic group spanning the entire genus intermitted by strains lacking that gene (Additional file 9A). Overall, *Clostridium* XIVa *cutC* protein sequences form a monophyletic clade (Additional file 1), where phylogenetic relations, with a few exceptions such as *Clostridium phytofermentans*, *Clostridium* sp. AT5, and *Dorea* sp. 5-2, resemble those derived from 16S rRNA gene sequence analysis (Additional file 9B) suggesting a close linkage between host phylogeny and *cutC* gene evolution. This indicates that gene loss rather than horizontal gene transfer is driving the disparate ability to transform choline into TMA within strains of this genus.

Results of global feature analysis based on FIGfams largely reflected 16S rRNA gene phylogeny where *cutC*-containing strains span the entire dendrogram and are dominated by three separate groups (Additional file 10A). *C. phytofermentans*, *Clostridium* sp. AT5, and *Dorea* sp. 5-2 are clustering apart displaying ~55% functional similarity with members of the three main groups, while *Eubacterium* sp. strain AB3007, together with three additional strains, forms the outgroup sharing less than half of FIGfams with other strains. More specific functional predictions were performed based on IMG’s phenotype analysis as well as by screening genomes for carbohydrate-active enzymes (CAZymes). The former analysis suggested that strains are auxotrophic for several amino acids (on average, 11 ± 2; Additional file 10B). Since complex polysaccharides that resist adsorption in the upper gastrointestinal tract form the major energy source for colon-living bacteria and several *Clostridium* XIVa strains are reported to grow on such compounds, their functional repertoire of CAZymes was of specific interest. All strains are predicted to exhibit CAZymes linked to the break-down of various diet and host-derived substrates (panel C in Additional file 10). Overall, strains are predicted to contain 101 ± 79 glycoside hydrolases (GHs), 41 ± 13 glycosyltransferases (GTs), 25 ± 17 carbohydrate-binding modules (CBMs), 26 ± 13 carbohydrate esterases (CEs), and 4 ± 4 polysaccharide lyases (PLs).

## Discussion

To analyze the human intestinal TMA-producing bacteria, we established a framework consisting of databases for the key genes of the major TMA-forming pathways and gene-targeted assays for their enumeration. Given that 16S rRNA gene sequence analysis cannot adequately predict the TMA-producing potential (Additional file 7) and metagenomic data lack depth to satisfactorily resolve this low abundant functional community, the assays developed here represent the most proper technique to investigate intestinal TMA-producing communities. With this in hand, we were able to quantify potential colonic TMA producers and gain detailed insights into their diversity.

Any comprehensive functional analysis of entire communities requires accurate definition of biochemical pathways and associated genes. Two distinct enzyme clusters involved in TMA production with the key genes *cutC* [6] and *cntA* [7] were previously described. A third enzyme, namely YeaW that is widespread in *E. coli*, was recently proposed as the central enzyme of a third major pathway for the conversion of carnitine to TMA [8]. However, the *yeaW* gene displays close sequence identity (74%) to the *cntA* model sequence from *A. baumannii* and is arranged in similar operons that additionally encode a reductase (*yeaX* and *cntB*, respectively) as well as a betaine/carnitine/choline transporter, and for both enzymes, promiscuity to transform carnitine and butyrobetaine at similar rates was shown (*yeaW/X* was reported to act on choline and to a lesser extent on betaine as well). In this study, we, hence, considered YeaW as belonging to the CntA cluster of enzymes as originally proposed by Zhu and colleagues [7]. Importantly, a recent report based on a genome-screening approach suggesting that numerous taxa contain both *cntA/B* and *yeaW/X* is misleading [11] as the database developed in this study unequivocally shows that the vast amount of genomes exhibits a single copy of this gene group.

The established databases present a comprehensive update of previous *cutC* [9] and *cntA* [7] gene sequence collections adding a multitude of new gene sequences. They will be regularly updated to serve as a resource for other researchers as well. Our gene-targeted assays encompass key genes of the two major TMA-synthesis pathways and target a broad range of distinct taxa. Primer coverage is thorough, and amplification as well as quantification of numerous distinct target sequences at similar levels was confirmed. Nevertheless, some sequences comprised in our databases are not expected to be amplified, as the *cntA* forward primer does not match a few *Betaproteobacteria*, and in the case of *cutC*, a few sequences observed in *Streptococcus* show ≥2 mismatches with the reverse primer. However, for both genes, metagenomic analysis suggested that sequences not targeted by our assays do not play an important role in the colonic TMA-producing communities as not a single read was related to any of those reference sequences. It should be noted that for amplification of functional genes, high degeneracy of primers is needed in order to ensure detection of the entire diversity. Since this can result in unspecific binding (in particular seen here for *cntA*), we suggest to quality control for every sample (see Additional file 4) in order to draw accurate conclusions on target abundance.

Target genes were detected in all samples suggesting that TMA producers constitute members of the core gut community, though at very low abundances. In the HMP dataset, 25% of samples were negative for *cutC/D* and *cntA/B* genes, which is most probably due to a lack of sequencing depth. The high diversities of possible TMA producers observed in fecal samples, in particular of *cutC* gene sequences, demonstrate that individual communities are characterized by vast functional redundancies where several taxa potentially contribute to the TMA pool. Sequences associated with three distinct phyla were detected, but the majority were associated with those observed in members of the *Clostridium* XIVa (and a specific *Eubacterium*). Although we detected competition patterns between related taxa, in particular between clusters a and b where either taxon dominated in specific samples (Fig. 2), co-occurrence of various phylogenetically close TMA-producing genes was observed in most samples. Functional predictions on respective reference genomes might explain those observations as they indicate broad, only partly overlapping ecological niches of individual strains. More specifically, our analyses indicated that *Clostridium* XIVa strains are characterized by overproportional rich carbohydrate-active enzyme (CAZyme) repertoires within the *Lachnospiraceae* [13] where, next to dietary matter, host-derived mucin might serve as a major nutrient source. Global feature analysis based on FIGfams suggested large functional differences between members of *Clostridium* XIVa. However, the bulk of obtained *cutC* sequences was distantly related to known references of this genus, and predictions presented here can, hence, only partially define functional characteristics of the TMA-producing community.

The observation of highly diverse genes potentially encoding TMA-forming enzymes has direct medical implications as it demonstrates the complexity of the TMA-synthesizing source and demands precision treatment where whole consortia rather than single bacterial taxa need to be targeted in order to restrict production of this health hazardous compound. Our analyses identified distinct groups of TMA-producing gene pools that are each dominated by a few sequence types, similar to enterotypes of global intestinal communities [14], which might guide the development of type-specific intervention strategies. It should be mentioned that major health-promoting butyrate producers previously classified as belonging to *Clostridium* XIVa such as *Eubacterium rectale* and *Roseburia* represent members of other genera that does not comprise any known CutC-encoding strains.

TMA formation via *cntA* represents a distinctive branch of TMA synthesis where, next to the enzyme’s distinct substrate spectrum compared with the choline TMA-lyase, ecophysiologic features of its key player, *E. coli*, are substantially different to those of the major *cutC*-exhibiting taxa. In particular, its facultative anaerobic nature, the lack of amino acid auxotrophies, and a restricted CAZyme repertoire (data not shown) demonstrate that this taxon thrives in distinct niches/environments compared with bacteria containing *cutC*. Thus, specific conditions that select against the latter bacteria might be compensated by an increase of taxa encoding CntA [7]. In fact, results of the two pathways did not correlate, which is highlighted by two samples derived from the HMP dataset that display exceptionally high amounts of *cntA*, but only minute *cutC* abundances (Fig. 4). This emphasizes the need to target the entire TMA-production potential of gut communities including all major pathways and taxa involved in order to obtain adequate functional assessments.

Samples used in this study derived from volunteers that differed substantially in various characteristics such as age, BMI, and health status (Additional file 5), which allowed insights into the TMA-producing community from a diverse population, but hindered revealing specific host factors that govern its proliferation. Furthermore, we only targeted the TMA-producing potential by quantifying respective gene abundances, and no investigations on gene expression/TMA production were performed limiting insights into actual functional activity. Reanalysis of the dataset provided by Karlsson and colleagues [12] indicated elevated abundances of potential TMA producers in atherosclerosis patients, though the methodology used, i.e., metagenomics, provided only limited insights and together with a low sample size it did not allow for extraction of statistically significant results (Additional file 8). Our developed assays are applicable in high throughput providing the basis for comprehensive studies investigating the link between gene abundance/expression, substrate availability, and TMA/TMAO serum concentrations in defined population groups, such as patients suffering from atherosclerosis. Next to characterizing the TMA-producing communities of risk groups, studies investigating specific factors governing the TMA-producing community are needed. In particular, diet is of interest as a correlation between dietary habits and production of TMAO was previously established. For instance, individuals on an omnivorous diet produced elevated TMAO levels in comparison to vegans or vegetarians following ingestion of l-carnitine [5]. Furthermore, patients suffering from trimethylaminuria, where TMA accumulates in various body fluids, are often treated by adapting to a vegan diet in order to limit the intake of dietary animal products that promote TMA production such as egg yolk and various meats [15]. The predicted profound amino acid auxotrophies of major players of the *cutC*-containing community (Additional file 10) suggest a link between the host diet, proliferation of TMA-producing strains, and concurrent TMA synthesis. Specifically, a nutrition that is rich in animal-derived products delivers both essential amino acids required for growth and high concentrations of the main TMA precursors choline and carnitine. In this context, it should be noted that it is unclear whether carnitine or choline itself are relevant selection factors for TMA-producing bacteria. Most bacteria, also those belonging to *Clostridium* XIVa as well as main *cntA*-containing taxa are able to utilize a multitude of energy sources in vivo where TMA precursors contribute, most probably, only minutely to the entire substrate pool, which is supported by the large CAZyme repertoires predicted for major TMA-producing taxa (see above).

## Conclusions

This study provides a diagnostic framework comprising comprehensive databases and gene-targeted assays that allowed, for the first time, the quantification and detailed characterization of the TMA-producing potential in human fecal samples. All subjects contained target genes where a multitude of potential TMA-producing taxa were revealed in most samples that, overall, clustered into distinct types characterized by highly abundant key players. Results of the developed assays together with presented functional predictions of major taxa involved provide crucial information for uncovering factors limiting growth of TMA producers in the gut and may assist the design of subject-specific therapies that prevent TMA synthesis and the development of disease.

## Methods

### Constructing the databases

Databases for the key genes *cutC* and *cntA* were built using a multiparametric approach similar to that previously described for butyrate producers [16]. A selection of protein reference sequences from literature [6, 7] (GenBank accession no.: *cutC*, ACL49259, ABB40076, EDU36695, EEU12078, EFJ62362, EEW38822, EEI47333; *cntA*, EFF74816, EGJ58898, EEW98385, EHL80614, EFK52065, EEW40126, EHL93080, EGQ26304) were aligned in Clustal Omega [17] and used to construct hidden Markov models (HMM) on full-length proteins via *hmmbuild* (default mode, HMMER 3.1b1, hmmer.org). Model seed sequences were realigned to the model using *hmmalign* (default mode) and manually checked in Jalview (jalview.org, v. 2.8.1) before rebuilding models based on the obtained alignments; this cycle was repeated three times to ensure construction of robust models. Subsequently, all 67,134 genomes provided by PATRIC (from https://www.patricbrc.org; April 2016) were screened (*hmmsearch* (--tblout)) for respective protein sequences. The top 3000 and 10,000 protein sequences (based on HMM score) from *cutC* and *cntA* searches, respectively, were collapsed (function *derep* [18]) and aligned to the HMM models (*hmmalign* in default mode). Partial gene sequences (<80% coverage to respective HMM models) were omitted, and conserved amino acid residues that were previously proposed as signature sites for the individual genes [6, 7] were determined manually for all sequences from the alignment files in Jalview. To infer phylogenetic distances to each top sequence of the two genes (based on HMM score from *hmmsearch* results), phylogenetic trees were constructed from the alignments (after conversion to fasta format using function *to-fasta* [18]) with the program FastTree (v. 2.1.8) [19] using the JTT+CAT model; pairwise distances between branch tips were determined using *cophenetic.phylo* function in R (v. 3.1.2) (package: ape, v. 3.4). Finally, genomes were screened for *cutD* and *cntB* (*hmmsearch*) using HMMs that were built (*hmmbuild*, *hmmalign* cycles as described above) from protein sequences that derived from the same genomes that contain the *cutC/cntA* seed sequences used above (to obtain seed sequences, reference sequences of *cutD* (ACL49260) and *cntB* (EEX03957) were BLASTED (blastp, v. 2.2.28+) against respective genomes; all seed sequences were in direct synteny (based on locus tag) with respective partner genes *cutC* and *cntA*; no *cntB* for *Sporosarcina newyorkensis* was found). Sequences were sorted based on HMM similarity scores, and low cutoffs at the end of obvious HMM score drops (HMM score of 51 and 26% of the top-scoring *cutD* and *cntB* sequences, respectively) were set to define putative *cutD*/*cntB* genes. Subsequently, their synteny with respective partner genes *cutC* and *cntA* was determined where genes separated by ≤10 genes (based on locus tag) were considered as syntenous. Due to the high consistency of all parameters investigated for *cutC*, HMM similarity cutoffs were chosen as the sole selection criterion resulting in 1114 candidate genes (454 unique proteins). For *cntA*, phylogeny was applied as an additional filter to the HMM score and 43 sequences (34 unique proteins; including *S. newyorkensis*) that displayed HMM similarities above the set cutoff were omitted yielding 6738 genomes that exhibit 6772 candidate genes (491 unique proteins). For taxonomic assignments, 16S rRNA gene sequences were collected from all genomes using a HMM model [20] and the longest sequence from each genome was subjected to the RDP classifier (--format = fixrank) applying a confidence interval of 80% [21]. For a few genomes that displayed only short sequences (<900 bp), the NCBI taxonomy was used as provided by PATRIC; for a few *Clostridium* XIVa-type strains, only short 16S gene sequences were obtained and RDP taxonomy was manually added. All genomes of candidates with respective taxonomy are listed in Additional file 2.

### Sample collection and DNA extraction

Human fecal samples from 50 volunteers were analyzed. No specific exclusion criteria based on health status of the volunteers were set. Their metadata is presented in Additional file 5. Many subjects were diagnosed with hypertension (28%), but none were known to suffer from atherosclerosis or severe cardiovascular disease. Samples were collected in a disposable kidney dish, and aliquots were transferred to 15-mL collection tubes (RNAlater solution added within a few hours after sampling (Sigma-Aldrich, Munich, Germany)) and stored at −80 °C. DNA was extracted using the FastDNA™ SPIN Kit for Soil (MP Biomedicals, Heidelberg, Germany) according to the manufacturer’s instruction and purified using the QIAquick PCR Purification Kit (Quiagen, Hilden, Germany). Nanodrop analysis (260/280 and 260/230 ratios) was performed as a crude quality control before analysis, and DNA concentrations were quantified with the 2030 Multilabel Plate Reader VICTOR X3 (Perkin Elmer, Waltham, MA, USA) using the Quant-iT Picogreen dsDNA assay kit (Invitrogen, Carlsbad, CA, USA).

### Development of gene-targeted assays

Gene abundances were measured by qPCR using degenerate primers (see Table 1) designed to target the majority of sequences from the constructed databases. We modified previously published primers [9] in order to amplify *cutC*, while primers targeting *cntA* were designed in-house (Table 1). All primers were synthesized and purified by Eurofins Genomics (Ebersberg, Germany). Amplification was performed with 30 ng of template DNA in a LightCycler 480 (Roche Diagnostics, Mannheim, Germany) using the HOT FIREPol EvaGreen qPCR Mix Plus (no Rox, Solis BioDyne, Tartu, Estonia) according to the manufacturer’s instructions. An initial 95 °C step for 15 min was followed by 40 cycles of denaturation at 95 °C for 45 s; annealing at 57 and 53 °C for *cutC* and *cntA*, respectively, for 45 s; and an extension step at 72 °C for 45 s deploying final primer concentrations of 2.5 and 0.75 μM for *cutC* and *cntA*, respectively. Melting curves were subsequently performed using the following program: 95 °C for 5 s, followed by 65 °C for 60 s, and a final continuous reading step of seven acquisitions per second between 65 and 97 °C. Standard curves were generated from dilutions of 10^6^ to 10^3^ genome copies where an equimolar mixture of DNA from *Clostridium hathewayi* (DSM 13479) and *Desulfovibrio desulfuricans* subsp. *aestuarii* (DSM 17919) was used for *cutC* quantifications, whereas *E. coli* K12 (DSM 10517) DNA served as the standard for *cntA*. Results are expressed as averages of three independent experiments, normalized to the total 16S rRNA gene copies from each sample using previously described primers [22] applying an annealing temperature of 55 °C.

**Table 1:** Primers targeting *cutC* and *cntA* as well as the V1-2 region of the 16S rRNA gene (with modified forward primer) are shown

	Forward primer (5′➔3′)	Reverse primer (5′➔3′)
*cutC*	TT**Y**GC**I**GG**I**TA**Y**CA**R**CC**N**TT	TG**N**GG**Y**TC**I**AC**R**CA**I**CCCAT
*cntA*	TA**Y**CA**Y**GCITGG**R**C**I**TT**Y**AA**R**CT	**R**CAGTG**R**TA**R**CA**Y**TC**S**A**KR**TAGTT**R**TC**R**AC
16S	AG**R**GTT**H**GAT**YM**TGGCTCAG	TGCTGCCTCCCGTAGGAGT

Bold letters highlight the degenerate bases within sequences (total combinations—cutC_F, 32; cutC_R, 16; cntA_F, 32; cntA_R, 512; 16S_F, 32; 16S_R, 1). Expected sizes of amplified products (excluding primers) are 275 bp (*cutC*) and 249 bp (*cntA*) based on references ACL49259 and EFK52065, respectively

In order to characterize the TMA-producing potential of microbial communities via sequencing, a three-step approach for library preparation was developed (Additional file 11) based on the dual index Illumina (San Diego, CA, USA) MiSeq sequencing procedure using the TrueSeq protocol. The genes *cutC* and *cntA* were amplified using the primers and amplification conditions as described above for qPCR (applying only 20 cycles). Products were purified (PureLink PCR Purification Kit; Invitrogen, Darmstadt, Germany) to remove primer dimers and 1 μL of the purified product served as template in a second PCR (seven cycles) using identical amplification conditions as in step one but applying target primers with a short overhang (Additional file 11). The PCR products were purified again (PureLink PCR Purification Kit; Invitrogen, Darmstadt, Germany) and subjected to a third amplification step that added the two indices and Illumina adapters to amplicons using PrimeSTAR HS DNA Polymerase (Takara, Otsu, Shigu, Japan) according to the manufacturer’s instructions. Thermocycling was performed as follows: 3 min at 95 °C, 10 s at 98 °C, 10 s at 55 °C, 45 s at 72 °C (×30), and 2 min at 72 °C. This step was specifically performed at high cycle numbers amplifying targets to adequate concentrations for sequencing as the deployed primers were non-degenerate minimizing possible amplification-derived bias. Amplicons were separated by gel electrophoresis, and the appropriate size was excised (cutC ~480, cntA ~460) and extracted with the QIAquick Gel Extraction Kit (Qiagen, Hilden, Germany). Obtained products were quantified by the Quant-iT Picogreen dsDNA assay kit as described above and pooled in equimolar ratios before sequencing on Illumina MiSeq (2 × 250 paired ends).

### Bioinformatic procedures for amplicon analysis

After quality filtering (Read Q25) and merging of raw paired-end reads [18], primers were trimmed off and sequences were subjected to FrameBot (v. 1.2, in default mode with option stat -t hist) analysis where all amplicons were aligned to reference sequences and frame-shift corrected as required [23]. The reference sets for each gene contained all unique sequences considered as *cutC* and *cntA* genes, respectively, as well as the 300 top-scoring unique protein sequences below the set HMM thresholds representing distinct sequences (see the “Constructing the databases” and “Results” sections; the 34 sequences derived from *Bacilli* located above the threshold though not considered as true *cntA* were included as well) as previously performed for butyrate producers [24]. This allowed a clear differentiation of amplicons that showed low similarity to reference sequences into those probably encoding enzymes for TMA production and others that are considered not to encode this function, which were not included in follow-up analyses. Sequences that displayed a stop codon were omitted. On average, 98.2% (*cutC*) and 92.5% (*cntA*) of all merged sequences passed these initial steps resulting in a mean of 40,915 ± 12,376 *cutC* sequences per sample (from 23,696 to 71,952) and 42,079 ± 7746 *cntA* sequences (from 28,471 to 52,687) derived from 50 and 12 samples, respectively. For *cntA*, sample 3 was omitted as >99% of obtained sequences (from 13,095) failed FrameBot analysis (this sample was also on the borderline of detection in qPCR and produced only a faint band during PCR amplification). For subsequent complete-linkage clustering (default mode [18]), the translated protein sequences were aligned to the HMM models and obtained alignments served as references to align the corresponding nucleotide sequences (function *align-nucl-to-prot* [18]), which were then subjected to clustering (at 90% nucleotide identity). All reference sequences from databases were trimmed to the amplicon region and included in this procedure. Only clusters that exhibit ≥5 sequence counts were considered, and the data was rarified to equal depth using the function *rarefy_even_depth* (*rngseed = TRUE*) from the R phyloseq package (v. 1.10.0) [25] before follow-up analyses. To retrieve the nucleotide identity of amplicons to references, sequences were BLASTED (blastn, v. 2.2.28+) against our databases and their identity was recorded. Heatmaps were created in R (v. 3.1.2) using the package gplots (v. 2.17.0) on logarithmic, relative abundance data (log(x + 1)). Neighbor-joining trees were constructed in MEGA (Molecular Evolutionar Genetics Analysis; version 6.0) applying 500 bootstraps using *p*-distance corrections and pairwise deletion of gaps and missing data. Co-occurrence analysis was performed as previously described [26] with data derived from the complete-linkage clustering result. Only significant correlations (*p* < 0.01) displaying a *ρ* ≥ 0.5 and a false-discovery correction *q*-value <0.01 between clusters that were detected in at least 50% of samples were considered for analysis. Non-metric multidimensional scaling analysis was performed in R (package: vegan, v. 2.3-4); cluster abundances were manually superimposed.

### Metagenomic analysis

Quality filtered sequence reads that were derived from 154 stool samples provided by the Human Microbiome Project (HMP [27]; data available at http://hmpdacc.org) were subjected to BLAST (*blastall*, -p blastn, v. 2.2.26) searches against the developed databases containing *cutC/D* and *cntA/B* genes. As for the amplicon analysis above, the reference set of each gene additionally included sequences that were below the set HMM threshold (500 top-scoring unique nucleotide sequences; for *cutD* and *cntB*, the threshold was defined by the lowest scoring gene that was syntenous with its partner gene; sequences derived from *Bacilli* located above the threshold though not considered as true *cntA* were included as well). Top hits (based on *e*-value) that covered ≥70 bp and displayed ≥70% identity to a reference were considered for analysis where both genes of each pathway had to be detected for a positive result. Median abundances of both genes were used to calculate pathway abundances expressed relative to the abundance of the housekeeping gene *rplB* (gene-length corrected), which had been extracted from all genomes based on a HMM model provided by RDP [28]. Only samples displaying both genes of a pathway were considered for analysis. Prior to taxonomic analysis of reads, the *cutC/D* reference genes were subjected to complete-linkage cluster analysis (90% identity on the nucleotide level [18] in default mode) as previously described [16], whereas sequences of *cntA/B* were taxonomically binned on the genus level. Median abundances of both genes of a pathway were used to calculate relative gene abundances associated with individual taxa. Furthermore, a recently developed gene-targeted metagenomic assembly tool that was specifically optimized for low target concentrations was used to screen samples (in default mode with a k-mer size of 45, a minimum contig size of 150 aa, and normalization of results to *rplB* abundance) [28]. As for the BLAST analysis, only samples containing both genes of a pathway were considered for analysis.

### 16S rRNA gene sequence profiling of samples

In order to compare results obtained with the gene-targeted approach with taxonomic data, the V1-2 region of the 16S rRNA gene was amplified from all samples. To ensure adequate amplification, including taxa that were previously underrepresented using conventional primers that target the V1-2 region such as *Bifidobacteria* [29], a modified forward primer was used (Table 1). One microliter of template DNA from extract was directly amplified using primers containing the overhangs (Additional file 11). Amplification was performed with the PrimeSTAR HS DNA Polymerase (Takara, Otsu, Shigu, Japan) according to the manufacturer’s instructions. Samples were denaturated for 3 min at 95 °C following 15 cycles of denaturation at 98 °C for 10 s, annealing at 58 °C for 10 s, and extension at 72 °C for 45 s. One microliter served as the template in a second 10-cycle PCR step adding the two indices and Illumina adapters to amplicons using amplification conditions as described above. Amplified products were purified, normalized, and pooled using the SequalPrep Normalization Plate and subjected to 250-bp paired-end Illumina MiSeq sequencing.

Raw reads were merged, filtered for a length of ≥250 bp, and subjected to the RDP classifier (--format = fixrank) [21]. Data was analyzed at the genus level. In order to obtain more detailed insights into the diversity of *Clostridium* XIVa-associated taxa, all sequences matching this taxon at a confidence interval ≥80% were additionally aligned to a reference set containing 16S rRNA gene sequences from all genomes classified as *Clostridium* XIVa (see above) using the RDP aligner (*pairwise-knn*) [18] and the top-scoring reference of each sequence was recorded.

### Genomics of *Clostridium* XIVa genomes

For global functional comparisons between strains, FIGfams (provided by PATRIC) were extracted from respective genomes; the cluster analysis based on binary Bray-Curtis dissimilarity calculations and construction of the associated dendrogram was performed in R (package: *vegan*, v. 2.3-4). Prediction of carbohydrate-active enzymes (CAZymes) was performed using family-specific HMMs provided by dbCAN [30] according to the developer’s instructions with default parameters. Amino acid auxotrophies were extracted from the phenotype analysis of corresponding entries on the Integrated Microbial Genomes database [31]. For all analyses, the genome of *Eubacterium* sp. AB3007 (1392487.3) was included.

## Additional files

Additional file 1: Neighbor-joining trees of all *cutC* (A) and *cntA* (B) protein sequences derived from the established databases. The phylogenetic position of the carrier is indicated on the class level by a color code except for *cntA* sequences from *Proteobacteria*, which are shown at the genus level. *CutC* sequences encoded by members of the genus *Clostridium* XIVa are indicated as well. On the right, neighbor-joining trees presented in a radial layout encompassing all unique protein sequences from our databases (*cutC* (C) and *cntA* (D) highlighted in gray) together with sequences below the set HMM cutoff threshold that were included in FrameBot analysis are shown. Sequences encoding a different function than *cutC* and *cntA* (based on uniprot (http://www.uniprot.org)) are shown in pink. For *cntA*, 34 unique proteins that were above the HMM similarity cutoff, but not included in our database due to their high phylogenetic distance to biochemically verified *cntA* sequences, are highlighted by the orange line (D). A: 1,2-propanediol dehydratase (WP_007885173); B: B12-independent glycerol dehydratase (AFH58722); C: Benzylsuccinate synthase alpha subunit (O87943); D: Formate acetyltransferase (P09373); E: 4-hydroxyphenylacetate decarboxylase (Q18CP5); a: choline monooxygenase precursor (AAB52509); b: choline monooxygenase (BAF93188); c: choline monooxygenase (CAE17671); d: 3-chlorobenzoate-3,4-dioxygenase oxygenase subunit (Q44256); e: toluate 1,2-dioxygenase large subunit (AAA26047); f: anthranilate dioxygenase large subunit (AAC34813). (PDF 612 kb)

Additional file 2: Taxonomies of all candidates exhibiting *cutC* (A) and *cntA* (B) based on RDP classification are given. Genomes where no/too short (<900 bp) 16S rRNA gene sequences were found are classified according to NCBI’s taxonomy. For a few genomes marked by “*”, names and respective functional genes suggest a distinct taxonomy as obtained by 16S rRNA gene sequence analysis and their original taxonomy from NCBI was kept. (XLSX 442 kb)

Additional file 3: Results of standard curves for *cutC* (A) and *cntA* (B) are shown, whereas amplification results of various short synthetic sequences containing the *cutC* (C) or *cntA* (D) primer target sequences are displayed below. A target copy number of 10^5^ was used (due to the short length of synthetic sequences compared with genomic DNA that was used for standard curves, the obtained values are below 10^5^). Error bars represent standard deviation on triplicate measurements. (PDF 254 kb)

Additional file 4: Quality controls after qPCR analyses. Products of *cutC* (A) and *cntA* (B) were separated by gel electrophoresis after qPCR to visually control amplification; melting curve analyses are shown below (results from different concentrations of the respective standards are highlighted in red). (PDF 527 kb)

Additional file 5: Metadata of volunteers providing fecal samples for the study. ABs: use of antibiotics within the last two weeks before sampling. (PDF 43 kb)

Additional file 6: Detailed diversity analysis of obtained *cutC* (A) and *cntA* (B) amplicons. Sequences were clustered on the nucleotide level over a range of identities from 99 to 90%. The *vertical*-axis shows the number of clusters (marked by white circle) at distinct clustering identities (*horizontal*-axis). At each clustering cutoff, two columns are depicted: the left column shows the cluster distribution, i.e., cluster presence in percentage of samples (binned into four distinct percentage categories), whereas the right column shows the relative abundance of clusters from each category. For instance, in panel A at a clustering cutoff of 5%, the third abundance category, i.e., present in 50–90% of samples (dark violet), comprises 2.5% of all (*n* = 1310) clusters (thus, 33 clusters) that contribute to 24.2% of total *cutC* sequences at that cutoff level. (PDF 47 kb)

Additional file 7: Relative abundance of major taxa previously reported to encode CutC (B) or CntA (C) from all 50 fecal samples analyzed, based on 16S rRNA gene sequence analysis. The key to the colors is displayed in the respective panels. All sequences classified as *Clostridium* XIVa were additionally binned into *cutC*-containing candidates (*cutC* +) and those lacking the gene (*cutC* −) based on closest match from alignments to all references of this genus. The fraction of *Enterobacteriaceae* that was classified as *Escherichia/Shigella* is displayed. The order of the samples is according to Fig. 2, and qPCR results of the gene-targeted assays are displayed in panel A to facilitate comparisons between analyses. Specific numeric labels give values exceeding the maxima of the axis. (PDF 56 kb)

Additional file 8: Abundance of *cutC* and *cntA* in healthy controls (C, *n* = 13) compared to symptomatic atherosclerosis patients (P, *n* = 12). Obtained *p* values (Mann-Whitney *U* test) are indicated. Raw metagenomic data from reference 12 was downloaded, subjected to adapter trimming (program trimmomatic from http://www.usadellab.org) and quality filtering using the program fastq_quality_filter (-q 30 -p 50) from the FASTX-Toolkit (http://hannonlab.cshl.edu/fastx_toolkit/), and reads were subsequently BLASTed against the *cut/cnt* gene databases developed here as described in the “Methods” section. (PDF 34 kb)

Additional file 9: Phylogenetic relationship (neighbor-joining trees) of all members of the *Clostridium* XIVa cluster based on 16S rRNA gene sequences (A) and *cutC* protein sequences (B). *cutC*-containing strains are highlighted in red in panel A. Bootstrap values >80% are represented as black circles. *Eubacterium* sp. AB3007 was included in the analysis. (PDF 634 kb)

Additional file 10: Functional analysis of all *Clostridium* XIVa strains and *Eubacterium* sp. strain AB3007. Panel A shows the global functional analysis of strains based on presence/absence (binary Bray-Curtis dissimilarity) using FIGfams; *cutC*-containing strains are highlighted in red. The heatmap below (B) displays amino acid auxotrophies and prototrophies for strains using IMG’s phenotype analysis; the IUPAC amino acid code is used. Panel C illustrates the most abundant CAZymes of individual families (GH: glycoside hydrolases; GT: glycosyltransferase; CBM: carbohydrate-binding modules; CE: carbohydrate esterases; PL: polysaccharide lyases) where “H” represents catabolic genes associated with degradation of host substances (based on CAZypedia). (PDF 1988 kb)

Additional file 11: Schematic view of the three-step library preparation procedure developed for Illumina sequencing of *cutC* and *cntA* amplicons. For 16S rRNA gene amplification, a two-step procedure was applied omitting the first target-enrichment step. The overhang is part of the sequencing primer sites and consists of 18 and 20 bp that are fused to the 5′ end of the forward and reverse primers, respectively. (PDF 34 kb)

## References

[1] Wang Z, Klipfell E, Bennett BJ, Koeth R, Levison BS, Dugar B (2011). Gut flora metabolism of phosphatidylcholine promotes cardiovascular disease. Nature.

[2] Tang WHW, Wang Z, Levison BS, Koeth RA, Britt EB, Fu X (2013). Intestinal microbial metabolism of phosphatidylcholine and cardiovascular risk. N Engl J Med.

[3] Trøseid M, Ueland T, Hov JR, Svardal A, Gregersen I, Dahl CP (2015). Microbiota-dependent metabolite trimethylamine-N-oxide is associated with disease severity and survival of patients with chronic heart failure. J Intern Med.

[4] Stubbs JR, Ocque AJ, Zhang S, Johnson C, Kimber C, House JA (2015). Serum trimethylamine-N-oxide is elevated in CKD and correlates with coronary atherosclerosis burden. J Am Soc Nephrol.

[5] Koeth R, Wang Z, Levison BS, Buffa J, Org E, Sheehy BT (2013). Intestinal microbiota metabolism of L-carnitine, a nutrient in red meat, promotes atherosclerosis. Nat Med.

[6] Craciun S, Balskus EP (2012). Microbial conversion of choline to trimethylamine requires a glycyl radical enzyme. Proc Natl Acad Sci U S A.

[7] Zhu Y, Jameson E, Crosatti M, Schäfer H, Rajakumar K, Bugg TDH (2014). Carnitine metabolism to trimethylamine by an unusual Rieske-type oxygenase from human microbiota. Proc Natl Acad Sci U S A.

[8] Koeth RA, Levison BS, Culley MK, Buffa JA, Wang Z, Gregory JC (2014). γ-Butyrobetaine is a proantherogenic intermediate in gut microbial metabolism of L-carnitine to TMAO. Cell Metab.

[9] Campo AM, Bodea S, Hamer H (2015). Characterization and detection of a widely distributed gene cluster that predicts anaerobic choline utilization by human gut bacteria. MBio.

[10] Romano KA, Vivas EI, Amador-Noguez D, Rey FE (2015). Intestinal microbiota composition modulates choline bioavailability. MBio.

[11] Falony G, Vieira-Silva S, Raes J (2015). Microbiology meets big data: the case of gut microbiota-derived trimethylamine. Annu Rev Microbiol.

[12] Karlsson FH, Fåk F, Nookaew I, Tremaroli V, Fagerberg B, Petranovic D (2012). Symptomatic atherosclerosis is associated with an altered gut metagenome. Nat Commun.

[13] Cantarel BL, Lombard V, Henrissat B (2012). Complex carbohydrate utilization by the healthy human microbiome. PLoS One.

[14] Arumugam M, Raes J, Pelletier E, Le Paslier D, Yamada T, Mende DR (2011). Enterotypes of the human gut microbiome. Nature.

[15] Christodoulou J (2012). Trimethylaminuria: an under-recognised and socially debilitating metabolic disorder. J Paediatr Child Health.

[16] Vital M, Howe A, Tiedje J (2014). Revealing the bacterial butyrate synthesis pathways by analyzing (meta) genomic data. MBio.

[17] McWilliam H, Li W, Uludag M, Squizzato S, Park YM, Buso N (2013). Analysis tool web services from the EMBL-EBI. Nucleic Acids Res.

[18] Cole JR, Wang Q, Fish JA, Chai B, McGarrell DM, Sun Y (2014). Ribosomal Database Project: data and tools for high throughput rRNA analysis. Nucleic Acids Res.

[19] Price MN, Dehal PS, Arkin AP (2010). FastTree 2—approximately maximum-likelihood trees for large alignments. PLoS One.

[20] Guo J, Cole JR, Zhang Q, Brown CT, Tiedje JM (2016). Microbial community analysis with ribosomal gene fragments from shotgun metagenomes. Appl Environ Microbiol.

[21] Wang Q, Garrity GM, Tiedje JM, Cole JR (2007). Naive Bayesian classifier for rapid assignment of rRNA sequences into the new bacterial taxonomy. Appl Environ Microbiol.

[22] Muyzer G, De Waal EC, Uitierlinden AG (1993). Profiling of complex microbial populations by denaturing gradient gel electrophoresis analysis of polymerase chain reaction-amplified genes coding for 16S rRNA. Appl Environ Microbiol.

[23] Wang Q, Quensen J, Fish J, Lee T (2013). Ecological patterns of nifH genes in four terrestrial climatic zones explored with targeted metagenomics using FrameBot, a new informatics tool. MBio.

[24] Vital M, Gao J, Rizzo M, Harrison T, Tiedje JM (2014). Diet is a major factor governing the fecal butyrate-producing community structure across Mammalia, Aves and Reptilia. ISME J.

[25] McMurdie PJ, Holmes S (2013). Phyloseq: an R package for reproducible interactive analysis and graphics of microbiome census data. PLoS One.

[26] Williams RJ, Howe A, Hofmockel KS (2014). Demonstrating microbial co-occurrence pattern analyses within and between ecosystems. Front Microbiol.

[27] HMP (2012). A framework for human microbiome research. Nature.

[28] Wang Q, Fish JA, Gilman M, Sun Y, Brown CT, Tiedje JM (2015). Xander: employing a novel method for efficient gene-targeted metagenomic assembly. Microbiome.

[29] Walker AW, Martin JC, Scott P, Parkhill J, Flint HJ, Scott KP (2015). 16S rRNA gene-based profiling of the human infant gut microbiota is strongly influenced by sample processing and PCR primer choice. Microbiome.

[30] Yin Y, Mao X, Yang J, Chen X, Mao F, Xu Y (2012). DbCAN: a web resource for automated carbohydrate-active enzyme annotation. Nucleic Acids Res.

[31] Markowitz VM, Chen IMA, Palaniappan K, Chu K, Szeto E, Grechkin Y (2012). IMG: the Integrated Microbial Genomes database and comparative analysis system. Nucleic Acids Res.

