# Supplementary figures and images for: Uncovering the trimethylamine-producing bacteria of the human gut microbiota
Source: Microbiome. 2017 May 15;5:54. doi: 10.1186/s40168-017-0271-9 (PMC5433236; doi:10.1186/s40168-017-0271-9)

**A**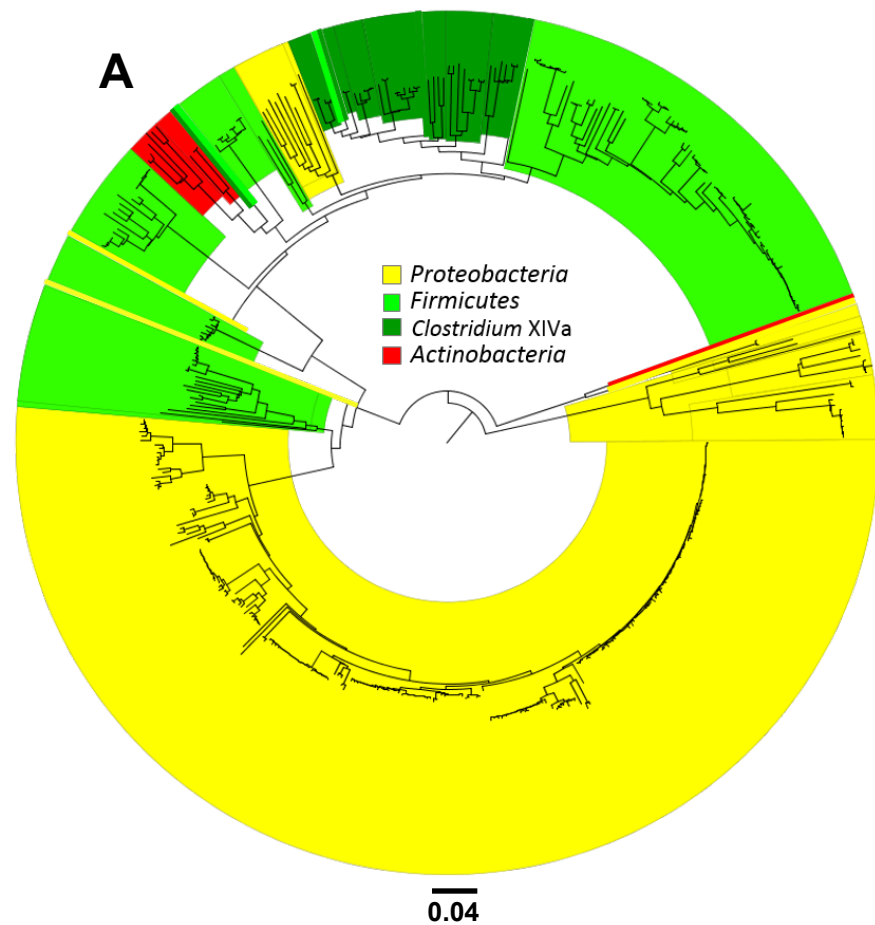**C**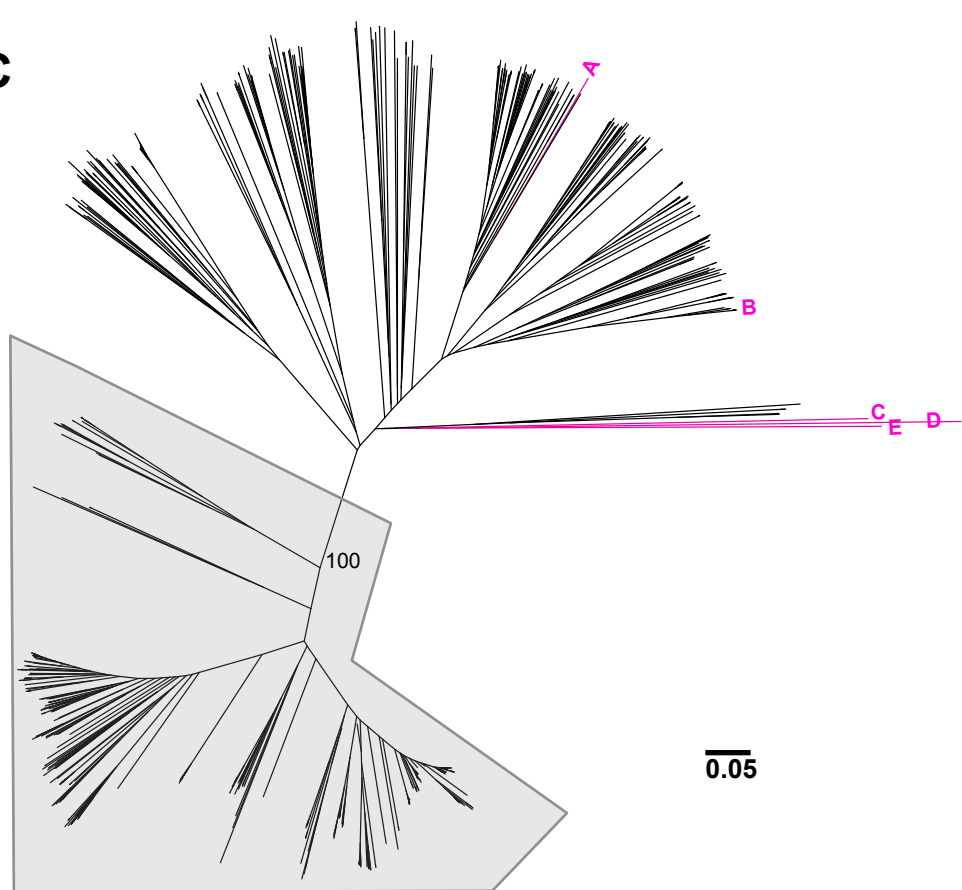**B**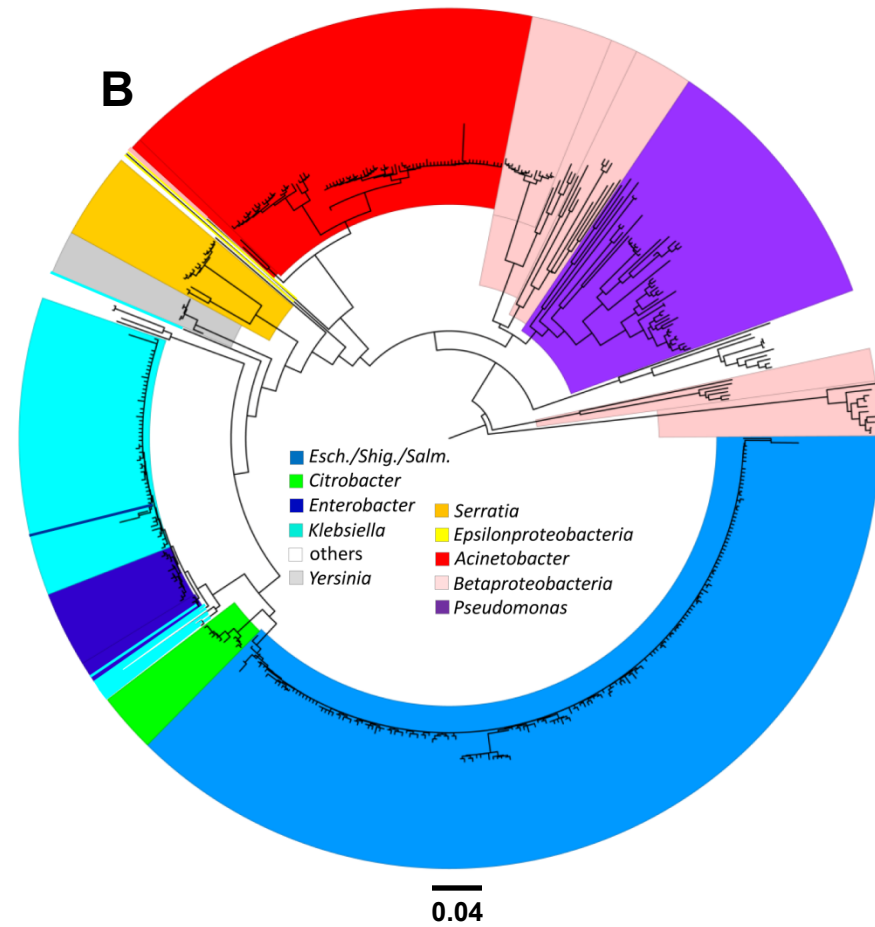**D**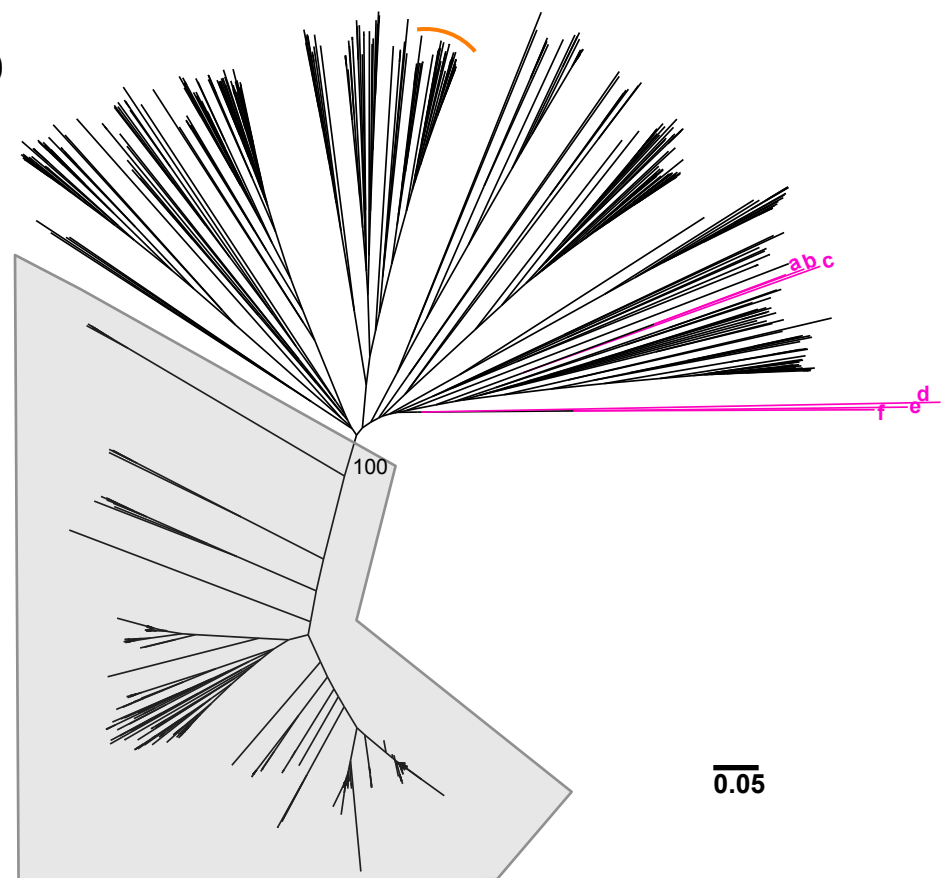

Supplement: Supplementary file 1 — Neighbor-joining trees of all cutC (A) and cntA (B) protein sequences derived from the established databases. The phylogenetic position of the carrier is indicated on the class level by a color code except for cntA sequences from Proteobacteria, which are shown at the genus level. CutC sequences encoded by members of the genus Clostridium XIVa are indicated as well. On the right, neighbor-joining trees presented in a radial layout encompassing all unique protein sequences from our databases (cutC (C) and cntA (D) highlighted in gray) together with sequences below the set HMM cutoff threshold that were included in FrameBot analysis are shown. Sequences encoding a different function than cutC and cntA (based on uniprot (http://www.uniprot.org)) are shown in pink. For cntA, 34 unique proteins that were above the HMM similarity cutoff, but not included in our database due to their high phylogenetic distance to biochemically verified cntA sequences, are highlighted by the orange line (D). A: 1,2-propanediol dehydratase (WP_007885173); B: B12-independent glycerol dehydratase (AFH58722); C: Benzylsuccinate synthase alpha subunit (O87943); D: Formate acetyltransferase (P09373); E: 4-hydroxyphenylacetate decarboxylase (Q18CP5); a: choline monooxygenase precursor (AAB52509); b: choline monooxygenase (BAF93188); c: choline monooxygenase (CAE17671); d: 3-chlorobenzoate-3,4-dioxygenase oxygenase subunit (Q44256); e: toluate 1,2-dioxygenase large subunit (AAA26047); f: anthranilate dioxygenase large subunit (AAC34813). (PDF 612 kb) [file 40168_2017_271_MOESM1_ESM.pdf]

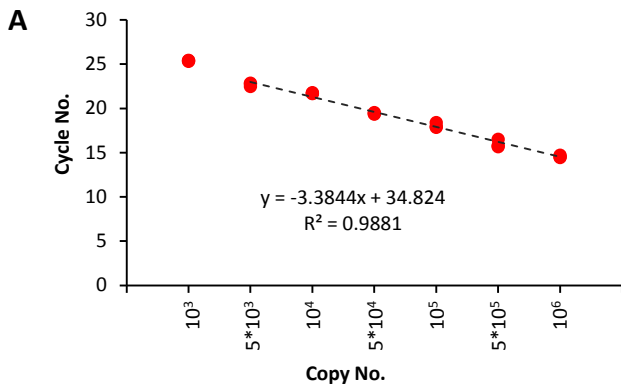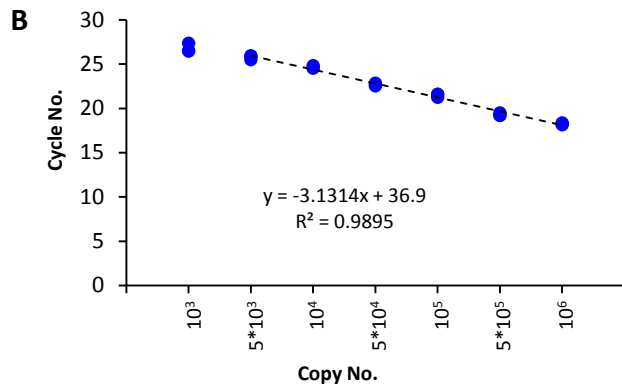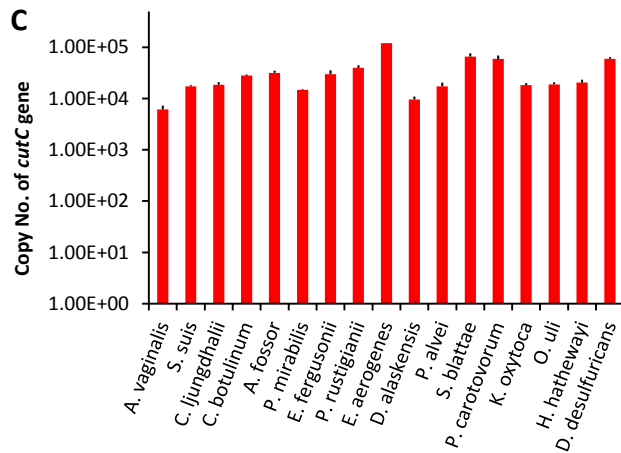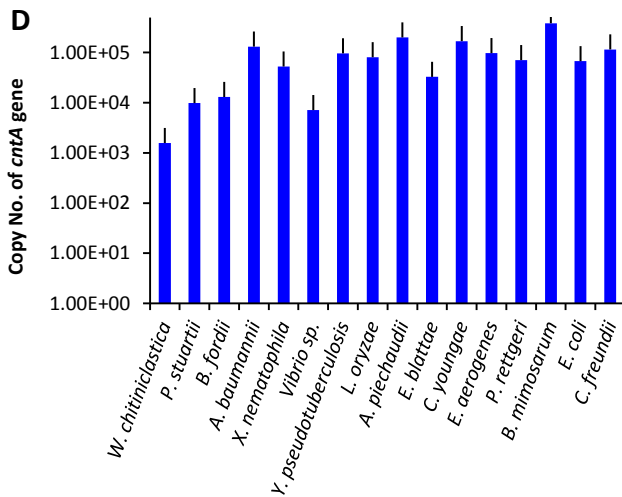

Supplement: Supplementary file 3 — Results of standard curves for cutC (A) and cntA (B) are shown, whereas amplification results of various short synthetic sequences containing the cutC (C) or cntA (D) primer target sequences are displayed below. A target copy number of 105 was used (due to the short length of synthetic sequences compared with genomic DNA that was used for standard curves, the obtained values are below 105). Error bars represent standard deviation on triplicate measurements. (PDF 254 kb) [file 40168_2017_271_MOESM3_ESM.pdf]

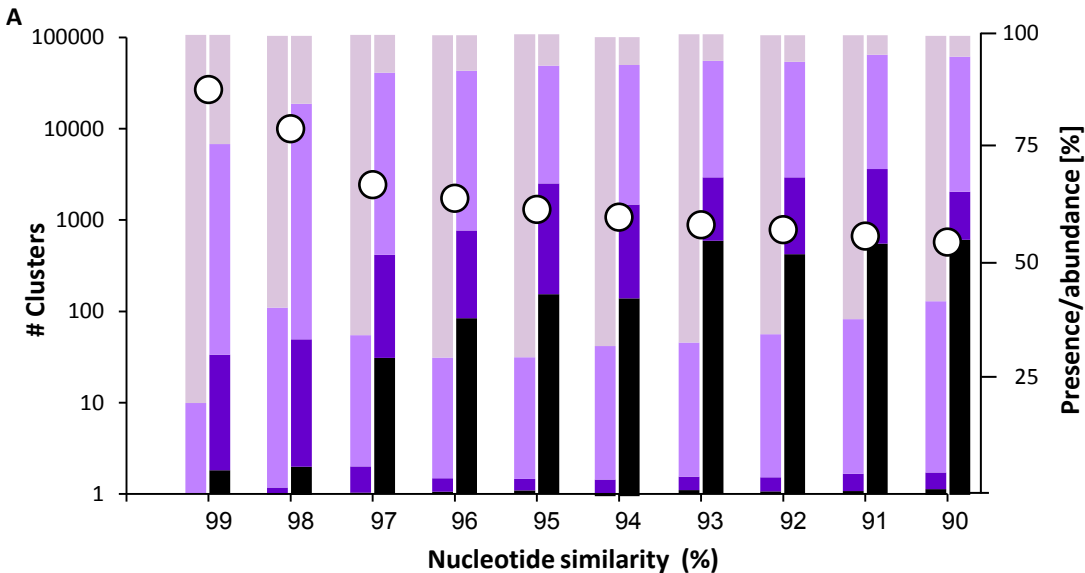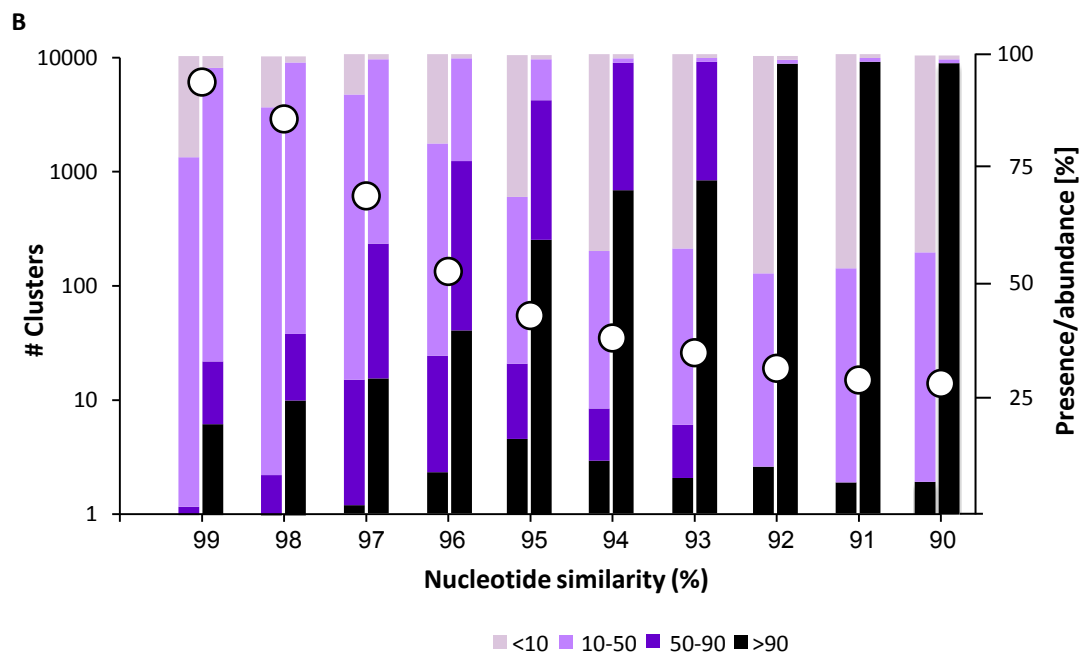

Supplement: Supplementary file 6 — Detailed diversity analysis of obtained cutC (A) and cntA (B) amplicons. Sequences were clustered on the nucleotide level over a range of identities from 99 to 90%. The vertical-axis shows the number of clusters (marked by white circle) at distinct clustering identities (horizontal-axis). At each clustering cutoff, two columns are depicted: the left column shows the cluster distribution, i.e., cluster presence in percentage of samples (binned into four distinct percentage categories), whereas the right column shows the relative abundance of clusters from each category. For instance, in panel A at a clustering cutoff of 5%, the third abundance category, i.e., present in 50–90% of samples (dark violet), comprises 2.5% of all (n = 1310) clusters (thus, 33 clusters) that contribute to 24.2% of total cutC sequences at that cutoff level. (PDF 47 kb) [file 40168_2017_271_MOESM6_ESM.pdf]

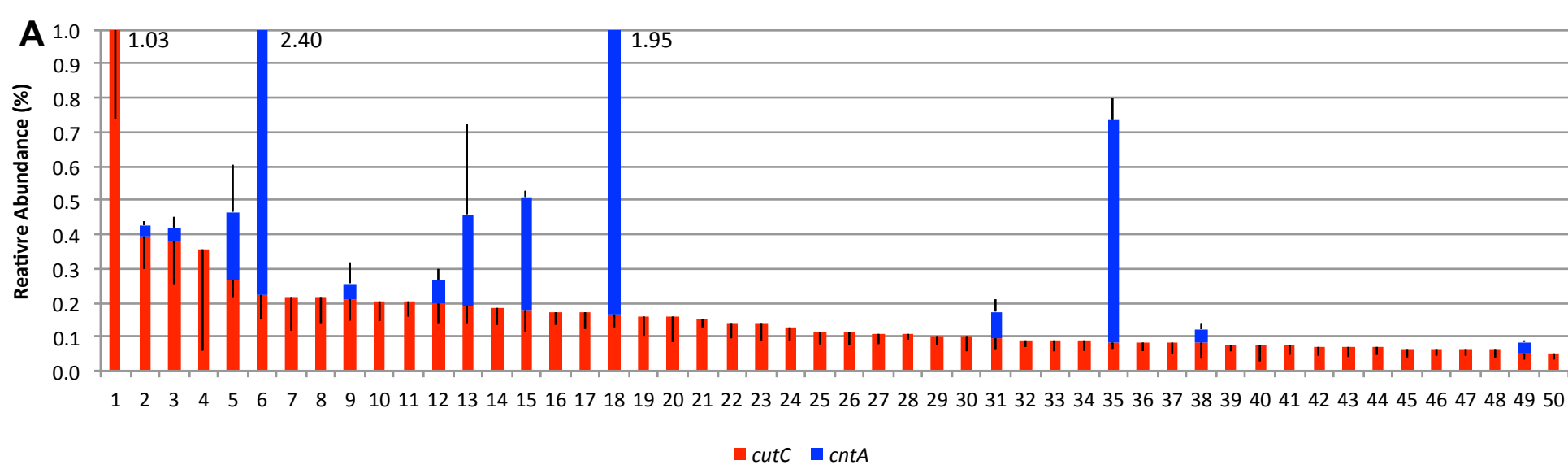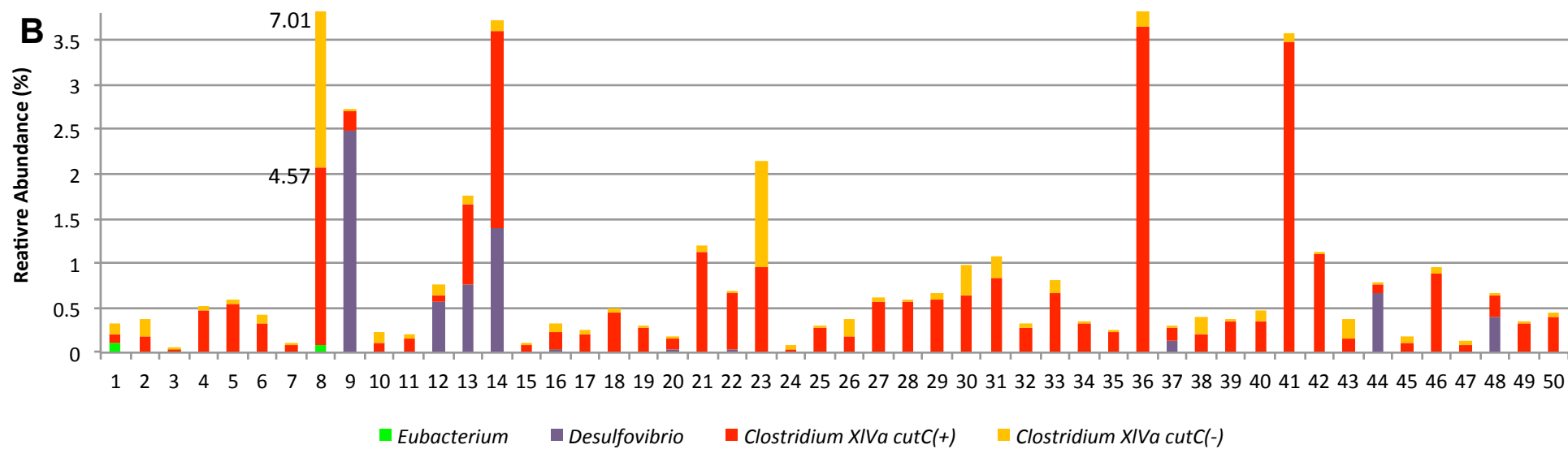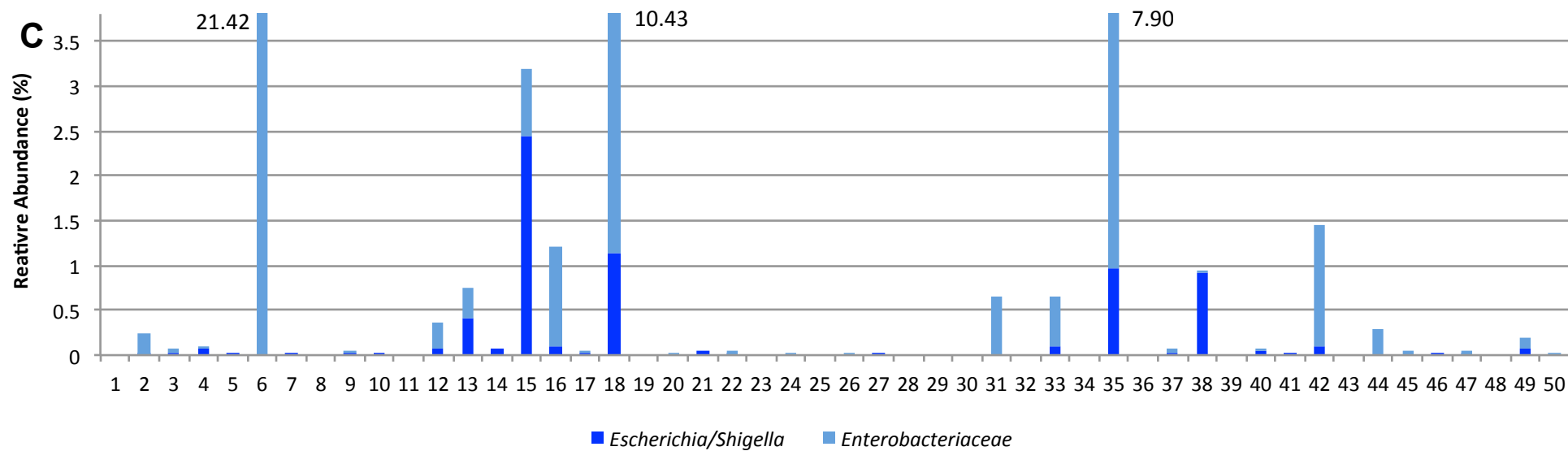

Supplement: Supplementary file 7 — Relative abundance of major taxa previously reported to encode CutC (B) or CntA (C) from all 50 fecal samples analyzed, based on 16S rRNA gene sequence analysis. The key to the colors is displayed in the respective panels. All sequences classified as Clostridium XIVa were additionally binned into cutC-containing candidates (cutC +) and those lacking the gene (cutC −) based on closest match from alignments to all references of this genus. The fraction of Enterobacteriaceae that was classified as Escherichia/Shigella is displayed. The order of the samples is according to Fig. 2, and qPCR results of the gene-targeted assays are displayed in panel A to facilitate comparisons between analyses. Specific numeric labels give values exceeding the maxima of the axis. (PDF 56 kb) [file 40168_2017_271_MOESM7_ESM.pdf]

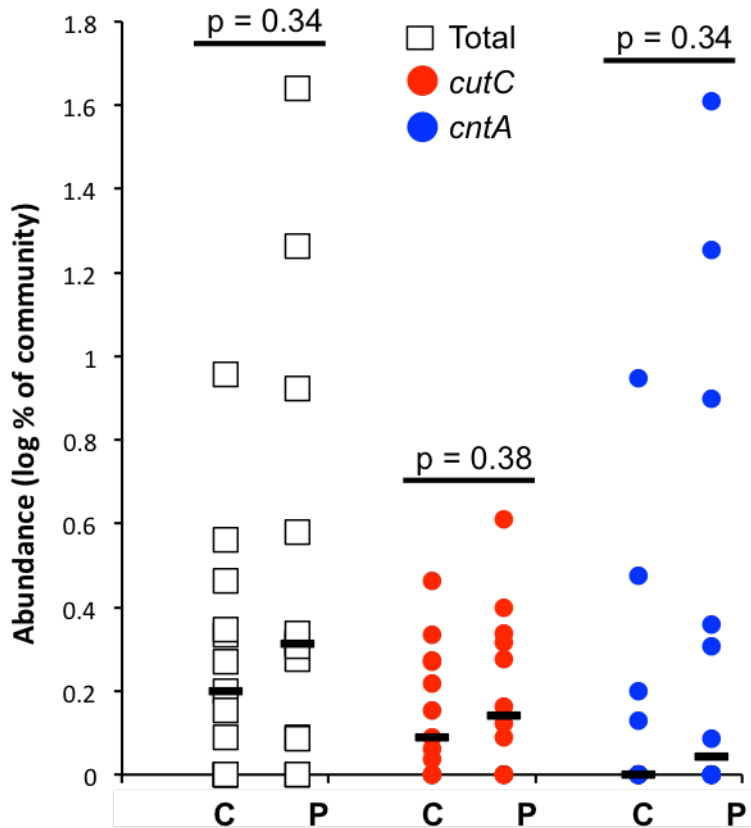

Supplement: Supplementary file 8 — Abundance of cutC and cntA in healthy controls (C, n = 13) compared to symptomatic atherosclerosis patients (P, n = 12). Obtained p values (Mann-Whitney U test) are indicated. Raw metagenomic data from reference 12 was downloaded, subjected to adapter trimming (program trimmomatic from http://www.usadellab.org) and quality filtering using the program fastq_quality_filter (-q 30 -p 50) from the FASTX-Toolkit (http://hannonlab.cshl.edu/fastx_toolkit/), and reads were subsequently BLASTed against the cut/cnt gene databases developed here as described in the “Methods” section. (PDF 34 kb) [file 40168_2017_271_MOESM8_ESM.pdf]

A

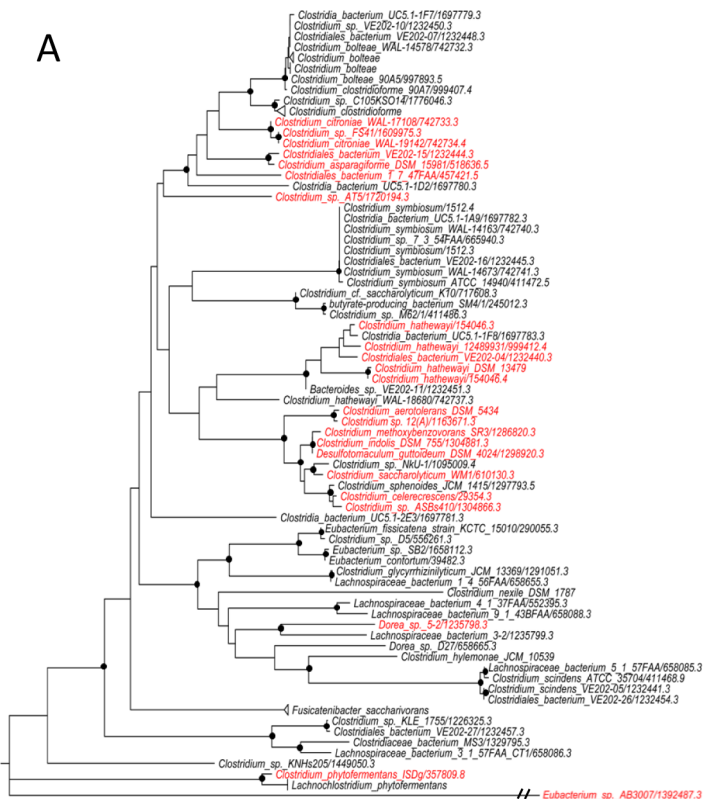

B

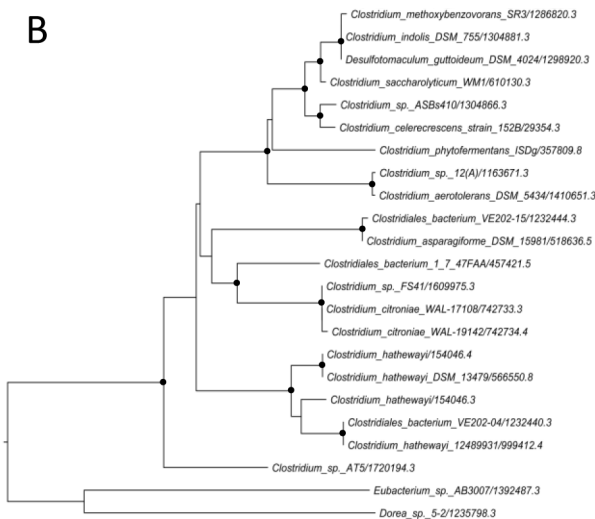

Supplement: Supplementary file 9 — Phylogenetic relationship (neighbor-joining trees) of all members of the Clostridium XIVa cluster based on 16S rRNA gene sequences (A) and cutC protein sequences (B). cutC-containing strains are highlighted in red in panel A. Bootstrap values >80% are represented as black circles. Eubacterium sp. AB3007 was included in the analysis. (PDF 634 kb) [file 40168_2017_271_MOESM9_ESM.pdf]

A – FIGfams analysis

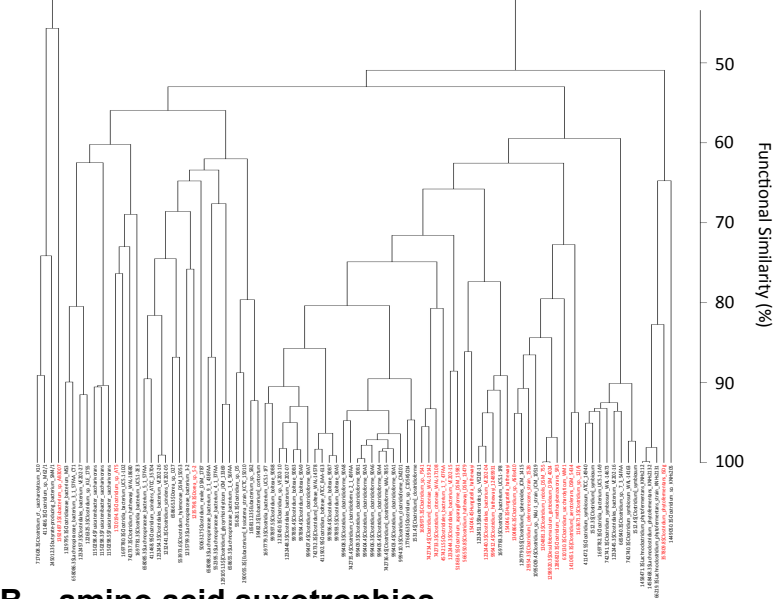

B – amino acid auxotrophies

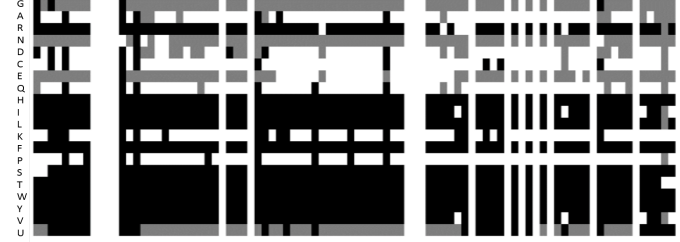

C - CAZymes

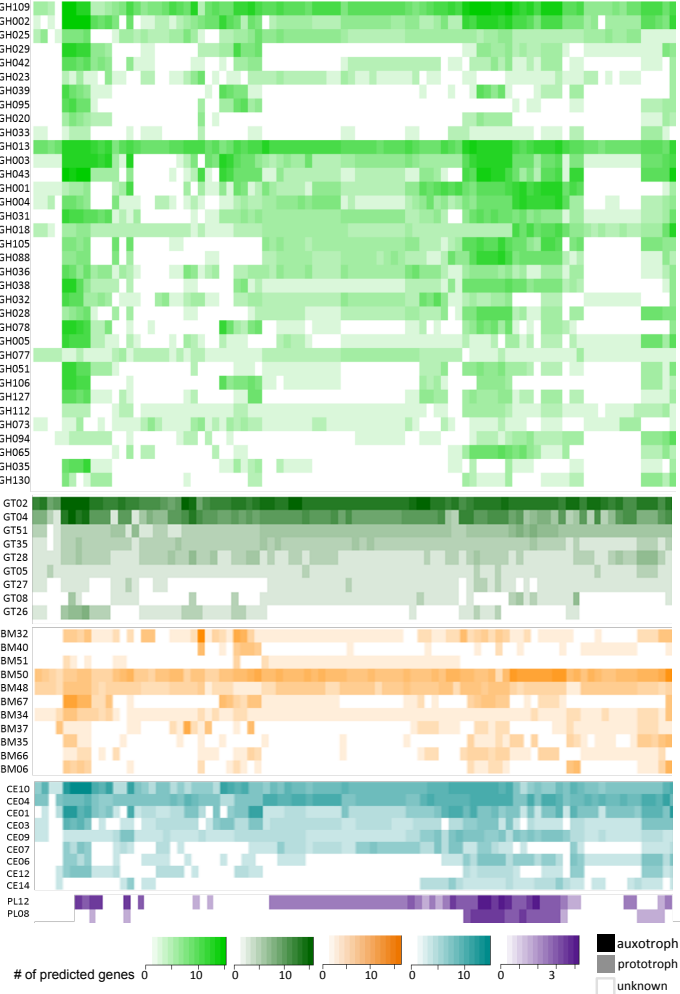

Supplement: Supplementary file 10 — Functional analysis of all Clostridium XIVa strains and Eubacterium sp. strain AB3007. Panel A shows the global functional analysis of strains based on presence/absence (binary Bray-Curtis dissimilarity) using FIGfams; cutC-containing strains are highlighted in red. The heatmap below (B) displays amino acid auxotrophies and prototrophies for strains using IMG’s phenotype analysis; the IUPAC amino acid code is used. Panel C illustrates the most abundant CAZymes of individual families (GH: glycoside hydrolases; GT: glycosyltransferase; CBM: carbohydrate-binding modules; CE: carbohydrate esterases; PL: polysaccharide lyases) where “H” represents catabolic genes associated with degradation of host substances (based on CAZypedia). (PDF 1988 kb) [file 40168_2017_271_MOESM10_ESM.pdf]
