# Supplementary material for: Uncovering the trimethylamine-producing bacteria of the human gut microbiota
Source: Microbiome. 2017 May 15;5:54. doi: 10.1186/s40168-017-0271-9 (PMC5433236; doi:10.1186/s40168-017-0271-9)

A Products of *cutC* qPCR analysis separated by gel electrophoresis

B Products of *cntA* qPCR analysis separated by gel electrophoresis

C Melting curves of *cutC* products after qPCR

D Melting curves of *cntA* products after qPCR

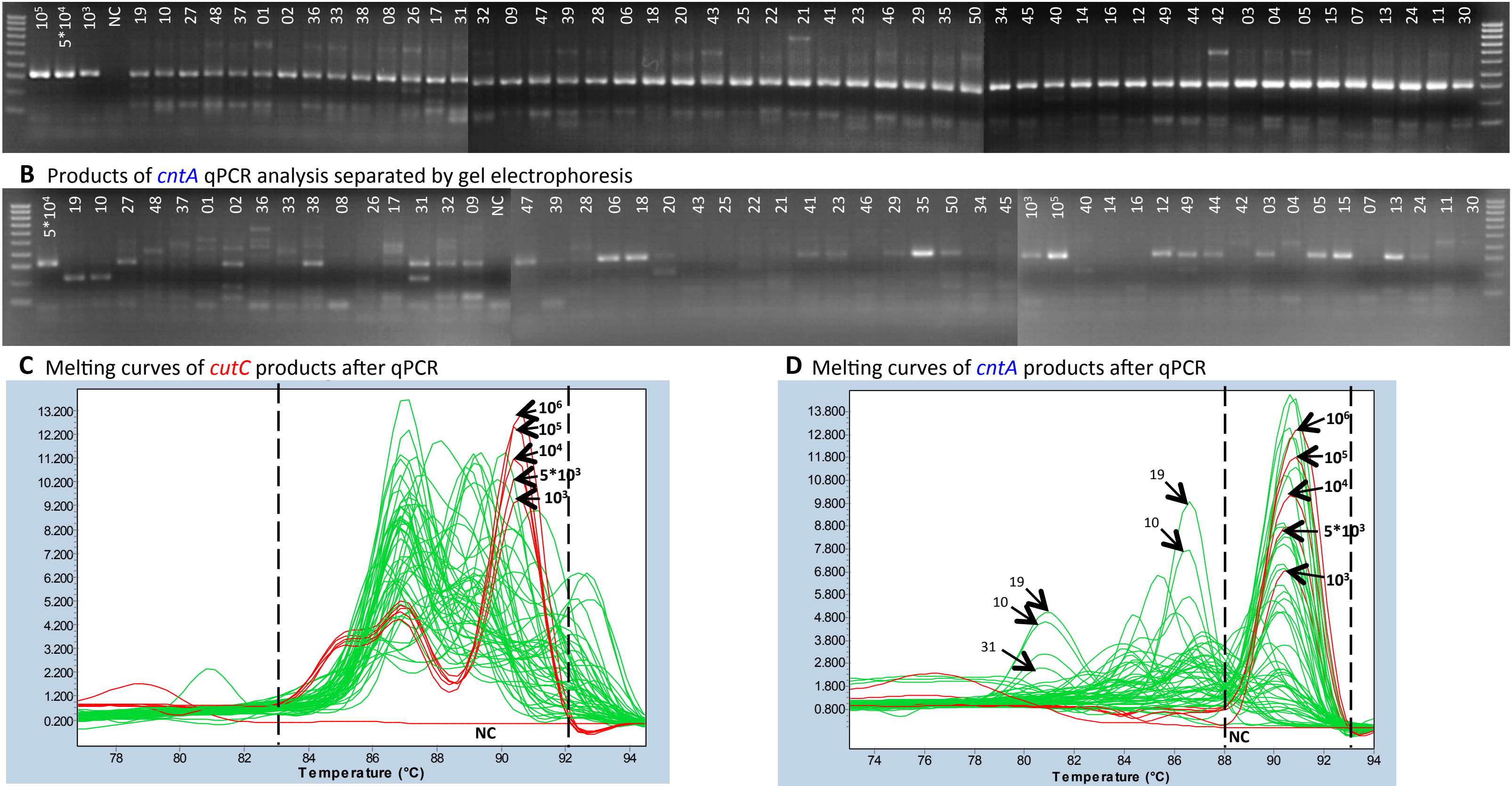

Supplement: Supplementary file 4 — Quality controls after qPCR analyses. Products of cutC (A) and cntA (B) were separated by gel electrophoresis after qPCR to visually control amplification; melting curve analyses are shown below (results from different concentrations of the respective standards are highlighted in red). (PDF 527 kb) [file 40168_2017_271_MOESM4_ESM.pdf]
