# Supplementary material for: Uncovering the trimethylamine-producing bacteria of the human gut microbiota
Source: Microbiome. 2017 May 15;5:54. doi: 10.1186/s40168-017-0271-9 (PMC5433236; doi:10.1186/s40168-017-0271-9)
Supplement: Supplementary file 5 — Metadata of volunteers providing fecal samples for the study. ABs: use of antibiotics within the last two weeks before sampling. (PDF 43 kb) [file 40168_2017_271_MOESM5_ESM.pdf]

	Sex	Age	BMI	Smoker	Disease(s)	ABs
**1**	f	32.8	21.9	-		-
**2**	m	54.3	26.6	+	Multiple sclerosis	-
**3**	f	35.1	24.8	-		-
**4**	f	45.7	26.2	+	Rest-less-legs, depression, psoriasis	-
**5**	m	32.9	27.6	-		-
**6**	m	58.9	36.6	-	Hypertonus	-
**7**	m	58.7	34.0	-	Hypercholesterolaemia, coronary artery disease	-
**8**	f	60.9	26.4	+	Hypertension	-
**9**	m	35.9	20.7	+	Ventricular extrasystoles	-
**10**	f	38.9	27.2	-		-
**11**	f	55.7	29.8	-		-
**12**	m	23.2	22.6	-		-
**13**	m	64.1	23.8	-		-
**14**	f	58.7	36.2	-	Status post stroke, diabetes, gout, hypertonus, peripheral artery disease	-
**15**	f	59.6	27.4	-		-
**16**	f	25.3	22.0	-		-
**17**	f	24.3	20.4	-	Hay fever	-
**18**	f	57.9	30.1	-	Depression, arthrosis	-
**19**	m	39.0	24.8	-	Hypertension	-
**20**	f	64.7	25.2	-		-
**21**	f	55.5	25.0	-		-
**22**	f	76.8	36.1	-	Hypertonus	-
**23**	f	63.9	27.3	-	Hypothyroidism, hypertonus	-
**24**	f	64.2	19.9	-	Hypertonus, glaucoma, urticaria	-
**25**	m	78.1	31.6	-	Hypertonus, prostatic hyperplasia	-
**26**	m	29.1	24.3	-		-
**27**	f	83.0	26.1	-	Athrosis, hypertonus	-
**28**	f	42.9	23.2	-		-
**29**	m	28.0	22.9	-		-
**30**	m	58.6	25.5	+		-
**31**	m	20.2	41.6	-		-
**32**	f	26.3	19.5	-		-
**33**	m	60.6	34.0	+	Hypertension	-
**34**	f	28.9	26.7	-		-
**35**	f	33.4	25.5	-		-
**36**	m	30.4	23.4	+		-
**37**	m	31.8	24.9	-		-
**38**	f	27.8	17.7	-		+
**39**	m	40.1	26.4	-		-
**40**	f	47.6	21.8	+	Hypercholesterolaemia, allergic rhinitis	+
**41**	m	61.7	29.4	-	Hypertonus	-
**42**	m	60.0	29.7	-	Hypertonus	+
**43**	m	69.3	28.0	-	Hypertonus	-
**44**	f	51.3	27.5	-	Rheumatism	-
**45**	f	58.5	29.1	-	Hypothyroidism	-
**46**	m	28.4	25.1	-		-
**47**	f	26.5	20.3	-	Hypothyreosis, hay fever	-
**48**	m	29.4	22.3	-		-
**49**	m	19.3	17.8	-		-
**50**	f	34.0	33.9	-		-
