# Supplementary material for: Uncovering the trimethylamine-producing bacteria of the human gut microbiota
Source: Microbiome. 2017 May 15;5:54. doi: 10.1186/s40168-017-0271-9 (PMC5433236; doi:10.1186/s40168-017-0271-9)
Supplement: Supplementary file 11 — Schematic view of the three-step library preparation procedure developed for Illumina sequencing of cutC and cntA amplicons. For 16S rRNA gene amplification, a two-step procedure was applied omitting the first target-enrichment step. The overhang is part of the sequencing primer sites and consists of 18 and 20 bp that are fused to the 5′ end of the forward and reverse primers, respectively. (PDF 34 kb) [file 40168_2017_271_MOESM11_ESM.pdf]

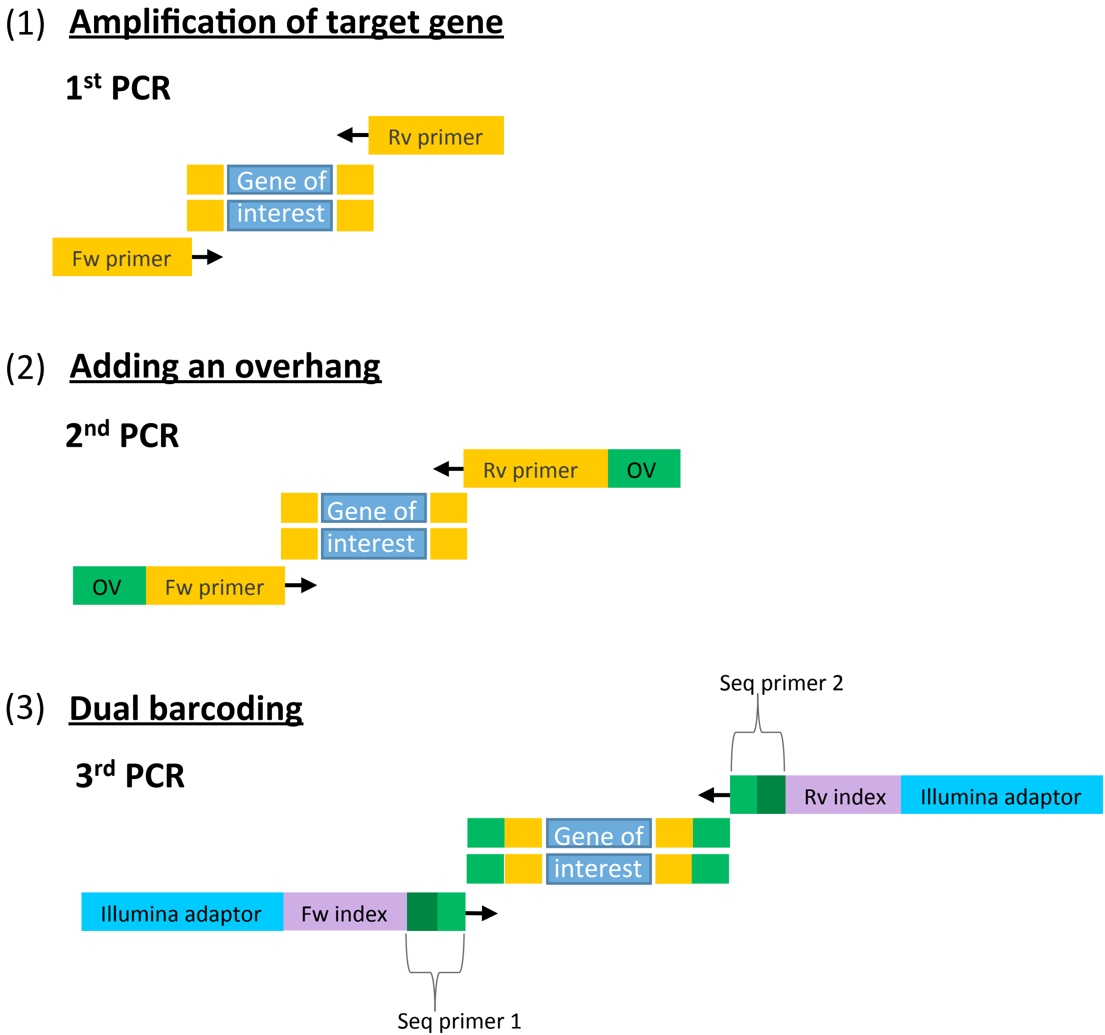
(1) Amplification of target gene
1st PCR
Rv primer
Gene of interest
Fw primer
(2) Adding an overhang
2nd PCR
Rv primer
OV
Gene of interest
OV
Fw primer
(3) Dual barcoding
3rd PCR
Seq primer 2
Rv index
Illumina adaptor
Gene of interest
Illumina adaptor
Fw index
Seq primer 1
